# Mechanisms underlying neutrophils adhesion to triple-negative breast cancer cells via CD11b-ICAM1 in promoting breast cancer progression

**DOI:** 10.1186/s12964-024-01716-5

**Published:** 2024-06-21

**Authors:** Chenghui Yang, Lili Li, Zhiqiang Ye, Anqi Zhang, Yunjia Bao, Xue Wu, Guohong Ren, Chao Jiang, Ouchen Wang, Zhen Wang

**Affiliations:** 1 Department of Breast Surgery, First Affiliated Hospital of Wenzhou Medical University, Wenzhou, 325000 P.R. China; 2 Department of Oncology, Second Affiliated Hospital, Zhejiang University School of Medicine, Hangzhou, 310009 P.R. China; 3 Key Laboratory of Tumor Microenvironment and Immune Therapy of Zhejiang Province, Second Affiliated Hospital, Zhejiang University School of Medicine, Hangzhou, 310009 P.R. China; 4 Department of Anesthesiology, First Affiliated Hospital of Wenzhou Medical University, Wenzhou, 325000 P.R. China; 5 First Clinical College of Wenzhou Medical University, Wenzhou, 325000 P.R. China; 6 Academy of Chinese Medical Sciences, Zhejiang Chinese Medical University, Hangzhou, 310005 P. R. China; 7 Department of Breast Surgery, Second Affiliated Hospital, Zhejiang University School of Medicine, Hangzhou, 310009 P.R. China

**Keywords:** Triple-negative breast cancer, Neutrophil, Tumor microenvironment, ICAM1

## Abstract

**Background:**

Triple-negative breast cancer (TNBC) is recognized as the most aggressive and immunologically infiltrated subtype of breast cancer. A high circulating neutrophil-to-lymphocyte ratio (NLR) is strongly linked to a poor prognosis among patients with breast cancer, emphasizing the critical role of neutrophils. Although the involvement of neutrophils in tumor metastasis is well documented, their interactions with primary tumors and tumor cells are not yet fully understood.

**Methods:**

Clinical data were analyzed to investigate the role of neutrophils in breast cancer. *In vivo* mouse model and *in vitro* co-culture system were used for mechanism researches. Blocking experiments were further performed to identify therapeutic agents against TNBC.

**Results:**

TNBC cells secreted GM-CSF to sustain the survival of mature neutrophils and upregulated CD11b expression. Through CD11b, neutrophils specifically binded to ICAM1 on TNBC cells, facilitating adhesion. Transcriptomic sequencing combined with human and murine functional experiments revealed that neutrophils, through direct CD11b-ICAM1 interactions, activated the MAPK signaling pathway in TNBC cells, thereby enhancing tumor cell invasion and migration. Atorvastatin effectively inhibited ICAM1 expression in tumor cells, and tumor cells with ICAM1 knockout or treated with atorvastatin were unresponsive to neutrophil activation. The MAPK pathway and MMP9 expression were significantly inhibited in the tumor tissues of TNBC patients treated with atorvastatin.

**Conclusions:**

Targeting CD11b-ICAM1 with atorvastatin represented a potential clinical approach to reduce the malignant characteristics of TNBC.

**Graphical Abstract:**

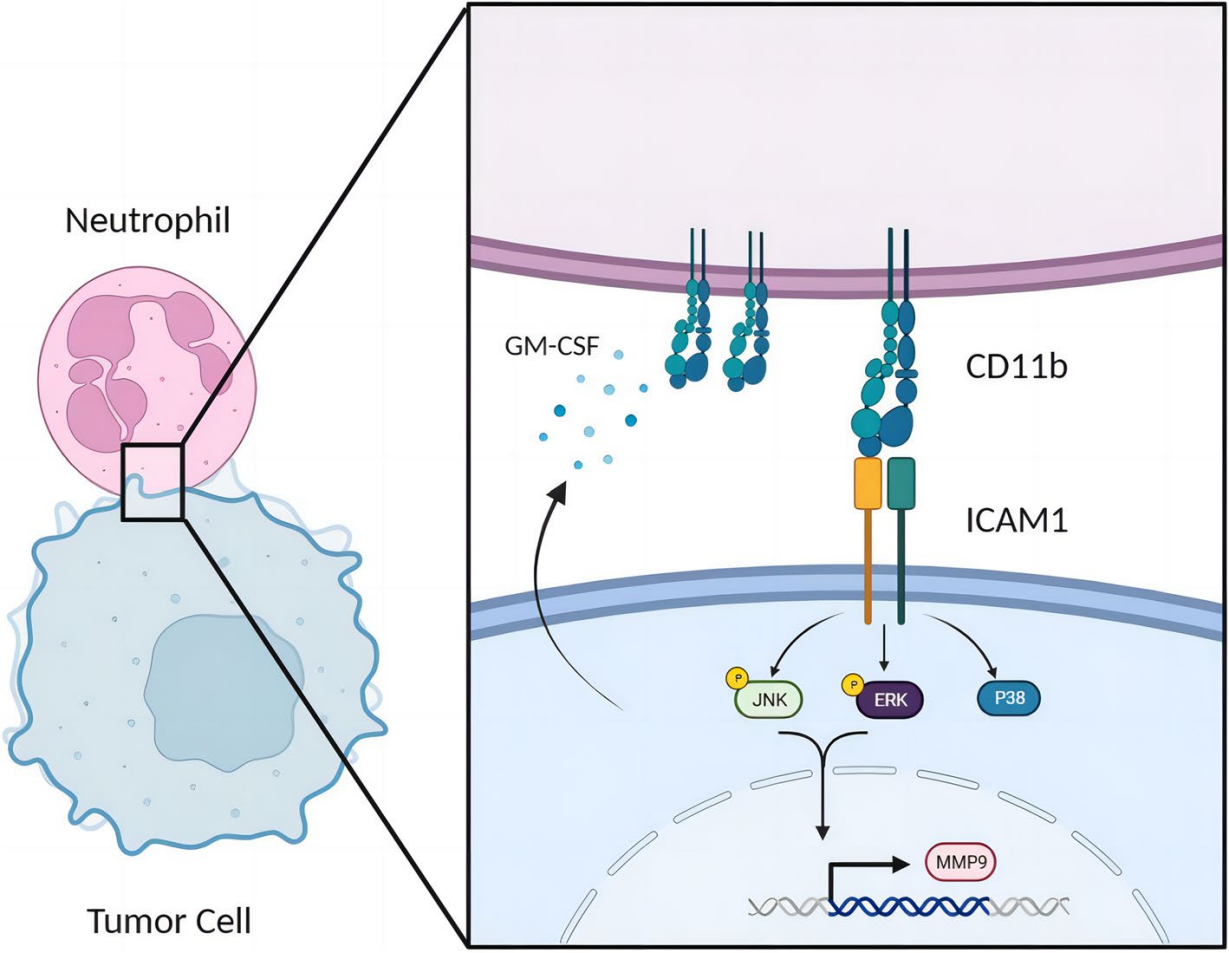

**Supplementary Information:**

The online version contains supplementary material available at 10.1186/s12964-024-01716-5.

## Background

Breast cancer, a prevalent malignancy among women worldwide, can be categorized into different molecular subtypes based on the expression of the estrogen receptor, progesterone receptor, human epidermal growth factor receptor 2 (Her2), and Ki-67 in tumor cells [1, 2]. Among the various subtypes, triple-negative breast cancer (TNBC) is the most aggressive subtype and is characterized by its high tumor invasiveness and the absence of targeted treatment options [3].

The transmembrane glycoprotein receptor ICAM1, a member of the family of cell adhesion molecules, serves as an adhesion protein. It is constitutively expressed at low levels in immune cells, endothelial cells, and epithelial cells but can be upregulated in response to inflammatory stimuli [4, 5]. Primarily, ICAM1 facilitates leukocyte recruitment to sites of inflammation. Additionally, it engages in immune system activation, cell signaling enhancement, and inflammatory responses by binding to the macrophage 1 antigen, lymphocyte function-related antigen-1, CD11a/CD18, and CD11b/CD18 [6, 7]. Although the role of ICAM1 in inflammation is well established, its function in tumors remains incompletely understood. Notably, ICAM1 expression levels are higher in TNBC than in the other breast cancer subtypes [8]. ICAM1 has been implicated in promoting TNBC bone metastasis by increasing apoptosis resistance through the TGF-β/SMAD signaling pathway [9]. Furthermore, it mediates the aggregation and migration of circulating tumor cells via intercellular homophilic interactions, thereby facilitating TNBC lung metastasis [10]. However, the impact of elevated ICAM1 expression on primary tumors has yet to be fully elucidated.

TNBC exhibits the highest mutation rate among all breast cancer types. Its high immunogenicity results in TNBC being the subtype with the most extensive immune cell infiltration [11]. Prior research has indicated that tumors with a higher proportion of tumor-infiltrating immune cells, referred to as "hot tumors", generally have a better prognosis and therapeutic response [12]. However, despite being the most immune-infiltrated subtype, TNBC has the poorest prognosis among breast cancers, which is closely related to the type of tumor-infiltrating immune cells [13]. 'Hot tumors' typically feature tumor-infiltrating lymphocytes (TILs), and studies have shown that TIL infiltration is positively correlated with a favorable prognosis in TNBC patients [14]. In contrast, a high neutrophil-to-lymphocyte ratio (NLR) is correlated with a poor prognosis in TNBC patients [15–18].

Given the prominent predictive role of the NLR in TNBC, the biological functions of neutrophils in TNBC have been further explored, particularly regarding neutrophil-mediated metastasis of triple-negative breast cancer. Studies, including our own, have shown that neutrophils can promote tumor cell proliferation, inhibit apoptosis, enhance angiogenesis, create an immunosuppressive microenvironment, and facilitate premetastatic niche formation by secreting myeloperoxidase (MPO), arginase 1 (ARG1), and neutrophil extracellular traps (NETs) [19–22]. Tumor-infiltrating neutrophils (TINs) interact with cancer cells within the tumor microenvironment (TME) [23]. The function of TINs remains debatable due to conflicting data from different cancer types [24]. In breast cancer, the role of direct interactions between TINs and tumor cells remains unclear.

In this study, we observed increased infiltration of neutrophils in primary TNBC tumors. Interestingly, these TINs were predominantly located around tumor cells exhibiting high ICAM1 expression. Through an *in vitro* co-culture system, we found that neutrophils exhibit greater adhesion to TNBC cells than to non-TNBC cells, suggesting that ICAM1 plays a role in tumor cell interactions with neutrophils. Mechanism investigations further revealed that GM-CSF facilitated the upregulation of CD11b expression in neutrophils, enabling their interaction with ICAM1 on tumor cells. CD11b-ICAM1 binding activated the MAPK signaling pathway in tumor cells, thereby promoting their migration and invasion. Additionally, we discovered that atorvastatin effectively inhibited ICAM1 expression in tumor cells. In an *in vivo* breast cancer mouse model, atorvastatin intervention significantly reduced ICAM1 expression on tumor cells, attenuated TINs infiltration, and downregulated the MAPK signaling pathway. These findings highlight the potential of atorvastatin as a therapeutic agent against TNBC.

## Methods

### Human sample

Peripheral blood (PB) and tissue paraffin sections were collected from the First Affiliated Hospital of Wenzhou Medical University. Blood samples were collected by licensed practical nurses at 6:00 a.m. before any treated intervention. Blood cells from Sodium Heparin Blood Collection Tube (BD vacuum™) were lysed for 15 minutes (BD Biosciences, Franklin Lakes, NJ, USA, #349202) and washed for subsequent flow cytometry analysis. All patients signed the informed consent, which was approved by the ethics review committee of the First Affiliated Hospital of Wenzhou Medical University.

### Cell culture

The MDA-MB-231, 4T1 and MCF7 breast tumor cell line were obtained from the Shanghai Institute of Cell Biology, Chinese Academy of Sciences (SIBS, Shanghai, China), and the EMT6 breast tumor cell line was obtained from the FuDan IBS Cell Center (FDCC, Shanghai, China). All cell lines were identified by STR profiling and incubated in a humidified incubator with 5% CO_2_ at 37°C. They were cultured in RPMI-1640 complete medium supplemented with 10% fetal bovine serum (Gibco, Grand Island, NY, USA) and 1:100 penicillin-streptomycin (Gibco).

For neutrophils culture, neutrophils were obtained according to the experimental requirements firstly and cultured in complete RPMI-1640 medium. For *in vitro* stimulation, Recombinant mouse G-CSF (BioLegend, San Diego, CA, USA, #574604, 50 ng/mL), recombinant mouse GM-CSF (BioLegend, #576304, 50 ng/mL), recombinant human G-CSF (BioLegend, #578604, 50 ng/mL), recombinant human GM-CSF (BioLegend, #572903, 50 ng/mL) or tumor cell culture supernatant was added into culture system. Cells were harvested at specific time stages and tested for cell survival using Annexin-PI (BD Bioscience, #556547).

## *3ICAM1* short hairpin RNA (shRNA) stable cell lines

4T1 and MDA-MB-231 cells were infected with *shICAM1* lentivirus or non-silencing control for 48 hours. Stable clones were selected using Blasticidin S hydrochloride. For 4T1 cells, the *ICAM1 shRNA* oligonucleotide sequences were as follows: *ICAM1 shRNA1*: 5′-CCGGATAACTGGACTATAATCATTCCTCGAGGAATGATTATAGTCCAGTTATTTTTTG-3′. *ICAM1 shRNA2*: 5′-CCGGCCAACTCTTCTTGATGTATTTCTCGAGAAATACATCAAGAAGAGTTGGTTTTTG-3′. *ICAM1 shRNA3*: 5′-CCGGACGCTGACTTCATTCTCTATTCTCGAGAATAGAGAATGAAGTCAGCGTTTTTTG-3′. For MDA-MB-231 cells, the *ICAM1 shRNA* oligonucleotide sequences were as follows: *ICAM1 shRNA1*: 5′-CCGGGATAGCCAACCAATGTGCTATTCAACTCGAGTTGAATAGCACATTGGTTGGCTATCTTTTTG-3′. *ICAM1 shRNA2*: 5′-CCGGCCGGTATGAGATTGTCATCATCTCGAGATGATGACAATCTCATACCGGTTTTTG-3′. *ICAM1 shRNA3*: 5′-CCGGGCCAACCAATGTGCTATTCAACTCGAGTTGAATAGCACATTGGTTGGCTTTTTG-3′. *Non-silencing shRNA* (*control shRNA*) was used as mock-transfected controls. Then *ICAM1* expression was verified by Real-Time qRT-PCR and western blot.

### Mice and *in vivo* intervention

Female wild-type BALB/c mice were provided by Slaccas Co. (Shanghai, China). All mice were housed in the specific pathogen-free conditions of Zhejiang Chinese Medical University Laboratory Animal Research Center. All mouse protocols and procedures were reviewed and approved by the Ethics Review Committee of the First Affiliated Hospital of Wenzhou Medical University.

In order to establish the 4T1 tumor-bearing mouse model, 8-week BALB/c mice were anesthetized with 0.8% sodium pentobarbital (80 mg/kg) intraperitoneal (*i.p.*) and inoculated with a suspension of 1x10^5^ 4T1 or EMT6 cells in the right fourth mammary fat pad.

For *in vivo* therapeutic treatment, after 4T1 cell inoculation in female BALB/c mice, Atorvastatin calcium (Macklin, Shanghai, China, #A797805) or ddH_2_O (Control group) was administered by oral gavage (10 mg/kg), five times per week for 4 weeks.

### Specimen acquisition and processing

Mouse bone marrow cells were obtained from the hind limbs and then filtered into single cells. Mouse primary tumors were cut into small pieces after excluding connective tissue and digested in digestion medium containing RPMI-1640 with 1 mg/mL collagenase IV (Sigma-Aldrich, St. Louis, MO, USA, #V900893) in a 37 ℃ shaker for 1-2 hour until digestion was completed. The single-cell suspension was further filtered through 40 μm nylon mesh (BD Falcon, #352340) to effectively remove impurities.

### Flow cytometry analysis and sorting

For cell surface marker staining, primary tumor cells, bone marrow cells were isolated as described previously and incubated with Zombie Red™ Fixable Viability Kit (BioLegend, #423109) for 30 minutes at room temperature (RT) and then washed with PBS. Cell suspension was then stained with Fc Receptor Blocking Solution (BioLegend, #101302 for mouse and #422302 for human) for 10 minutes and then with the following fluorochrome-conjugated mAbs for 30 minutes at 4 ℃: anti-CD45 (#103116 for mouse), anti-CD11b (#101216 for mouse), anti-EpCAM (#118206 for mouse), anti-CD54 (#116108 for mouse), anti-Ly6G (#127614 for mouse); anti-CD45 (#368516 for human), anti-CD11b (#301322 for human), anti-CD66b (#305104 for human), anti-CD54 (#353106 for human) (all from BioLegend). Isotype controls are applied as negative controls.

### Neutrophil magnetic isolation

Human neutrophil MACS isolation was performed using EasySep Direct Human Neutrophil Isolation Kit (STEMCELL, Vancouver, Canada, #19666). Briefly, peripheral blood was acquired, and 50 μL of Isolation Cocktail was added per 1 mL of whole blood. Then 50 μL RapidSphere magnetic nanobeads was added and incubated for 5 minutes at room temperature. Place the tube into the magnet (STEMCELL, #18001) and incubate for 5 minutes. the enriched cell suspension was poured into a new tube and the same volume of RapidSphere was added. The sample was incubated and placed into the magnet again. Repeated magnet isolation was operated after the suspension become faint yellow. The suspension was then centrifugated at 300g for 10 minutes and washed twice with complete RPMI-1640 medium.

Mouse neutrophil MACS isolation was performed using a Mouse Neutrophil Isolation Kit (Miltenyi, Bergisch Gladbach, Germany, #130-097-658). Briefly, single-cell suspensions from mice bone marrow or tumor tissue were acquired, and erythrocytes were lysed before magnetic labeling. Then, 50 μL of Neutrophil Biotin-Antibody Cocktail was added per 200 μL of cell suspension (5x10^7^ total cells) and incubated for 15 minutes on ice. After washing, 100 μL of Anti-Biotin MicroBeads was added per 400 μL of cell suspension. An LS column and a MidiMACS separator (Miltenyi) were used for subsequent magnetic sorting.

### *In vitro* cell co-culture

Mice neutrophils were isolated from the bone marrow (BM) of naive mice and human neutrophils were isolated from the blood of breast benign tumor patients, then obtained as above described, and been added into co-culture system (tumor cells: neutrophils = 1:20), seen in Fig. S2C.

For live cell imaging, GFP- expressing tumor cells were applied and neutrophils was stained with Dil dye (Beyotime, Shanghai, China, #C1036). Briefly, Dil Dye was diluted to 5 μM by RPMI-1640 medium and neutrophils was stained for 10 minutes, then washed with complete RPMI-1640 medium twice.

For *in vitro* blocking assay, inhibitors (10 ng/mL) including purified anti-GM-CSF (BioLegend, #50540 for mouse and #502319 for human), purified anti-CD11b (BioLegend, #101247) and atorvastatin was added according to the experiments.

For tumor cell RNA sequencing, after co-culture for the indicated times, the supernatant was aspirated and washed twice with PBS. Tryptase was added to the culture plate for digestion to obtain cell precipitation. Cell precipitation was resuspended using 100 μL MACS buffer, with 1 μL anti-CD45 antibody (BioLegend, #103103 for mouse and #304003 for human) added and incubated on ice for 15 minutes. Cell suspensions were washed once with cell staining buffer and resuspended with 90 μL MACS buffer again, and 10 μL Streptavidin Nanobeads (BioLegend, #480016) were added and incubated on ice for 15 minutes. The suspension was washed and resuspended with 1 mL MACS buffer. An LS column and a MidiMACS separator (Miltenyi) for subsequent magnetic sorting. The obtained negative cell suspension is the required tumor cells. RNA sequencing was operated as previous article described [22].

### Western Blot

Sorted tumor cells from co-culture system were harvested and lysed in pre-cooled RIPA Lysis Buffer (Beyotime, #P0013B) with a cocktail of protease and phosphatase inhibitor (Thermo Fisher, Waltham, Massachusetts, USA, #78445). A bicinchoninic acid (BCA) assay kit (Thermo Fisher, #23227) was used for protein concentration measurement. The proteins were separated by sodium dodecyl sulfatepolyacrylamide gel electrophoresis (SDS-PAGE) and then transferred onto a polyvinylidene difluoride (PVDF) membrane (Bio-Rad, Hercules, California, USA). After blocking with 5% (w/v) fat-free milk (BD Biosciences, #232100) at RT for 1 hour, the membrane was incubated with the corresponding primary antibodies overnight at 4 ℃ followed by the appropriate horseradish peroxidase (HRP)-conjugated secondary antibodies. Immunoreactive bands were identified using enhanced chemiluminescence (Thermo Fisher Pierce, #32109). Primary antibody, including Phospho-MAPK Family Antibody Sampler Kit (CST, Boston, MA, USA, #9910T), anti-P38 Rabbit mAb (CST, #8690T), SAPK/JNK Antibody (CST, #9252T), anti-ERK1+ERK2 antibody (Abcam, Cambridge, MA, USA, #ab184699), Anti-GAPDH (Huabio, Hangzhou, China, #SA30-01), anti-Beta Actin (Huabio, #B4-B2), HRP Conjugated Goat anti-Rabbit IgG Goat Polyclonal Antibody (Huabio, #HA1001), HRP Conjugated Goat anti-Mouse IgG Goat Polyclonal Antibody (Huabio, #HA1006), anti-ICAM1 Rabbit mAb (Abcam, #ab282575), anti-ICAM1 Rabbit mAb (Abcam, #ab222736) was applied. Secondary antibodies, including anti-mouse (1:5000, HuaBio, #G1006-1) and anti-rabbit (1:5000, HuaBio, #HA1001), were applied. Quantification of WB images was conducted by ImageJ software (version 1.48).

### Tissue immunofluorescence staining

Mouse tissue was obtained, fixed with 4% paraformaldehyde for 24 hours and then embedded in paraffin for sectioning. After dewaxing and dehydration, sections were incubated with a primary antibody overnight. A fluorophore-labeled secondary antibody was added and incubated for 2 hours at RT. Finally, the sections were stained with DAPI and imaged. Quantification of immunofluorescence images was conducted by ImageJ software. Primary antibody, including phospho-SAPK/JNK (Thr183/ Tyr185) Mouse mAb (CST, #9255S), anti-MMP9 Rabbit pAb (Abcam, #ab283575), anti-ICAM1 Rabbit mAb (Abcam, #ab282575), anti-ICAM1 Rabbit mAb (Abcam, #ab222736), Anti-Myeloperoxidase Rabbit mAb (Abcam, #ab208670), Anti-Ly6G Rabbit pAb (Servicebio, #GB11229-100) was applied.

### RNA isolation and quantitative real-time-PCR

Tissue samples were ground in liquid nitrogen before RNA isolating. Grounded tissue and cell samples were added directly to TRIzol Reagent (Thermo Fisher, #15596018CN) according to the manufacturer’s instructions. Concentration of RNA was examined by NanoDrop (Thermo Fisher). Total RNA was reverse-transcribed into cDNA using PrimeScript™ RT Master Mix (TaKaRa, Dalian, China, #RR036A), then amplified by TB Green Premix Ex Taq (TaKaRa, #RR420A) and detected by the 7500 Fast Real-Time system (Applied Biosystems). Data were processed using 7500 (V2.3) software (Applied Biosystems). Results were normalized based on housekeeping gene *β-actin* and then expressed as fold upregulation comparing with control. The sequences of the primers used were: Mouse *β-actin* forward: ATCGTGCGTGACATCAAAGA, Mouse *β-actin* reverse: ACAGGATTCCATACCCAAGAAG, mouse *ICAM1* forward: GTGATGCTCAGGTATCCATCCA, mouse *ICAM1* reverse: CACAGTTCTCAAAGCACAGCG, mouse *MMP9* forward: GCGTCGTGATCCCCACTTAC , mouse *MMP9* reverse: CAGGCCGAATAGGAGCGTC, mouse *MUC1* forward: GGCATTCGGGCTCCTTTCTT, mouse *MUC1* reverse: TGGAGTGGTAGTCGATGCTAAG, mouse *STAT3* forward: CAATACCATTGACCTGCCGAT, mouse *STAT3* reverse: GAGCGACTCAAACTGCCCT, mouse *LCN2* forward: GGGAAATATGCACAGGTATCCTC, mouse *LCN2* reverse: CATGGCGAACTGGTTGTAGTC, human *β-actin* forward: GGCTGTGCTATCCCTGTACG, human *β-actin* reverse: ACAGGATTCCATACCCAAGAAG, human *ICAM1* forward: TCCTCACCGTGTACTGGACT, human *ICAM1* reverse: GCCGGAAAGCTGTAGATGGT, human *MMP9* forward: AGACCTGGGCAGATTCCAAAC, human *MMP9* reverse: CGGCAAGTCTTCCGAGTAGT, human *MUC1* forward: TGCCGCCGAAAGAACTACG, human *MUC1* reverse: TGGGGTACTCGCTCATAGGAT, human *STAT3* forward: ACCAGCAGTATAGCCGCTTC, human *STAT3* reverse: GCCACAATCCGGGCAATCT, human *LCN2* forward: GACAACCAATTCCAGGGGAAG, human *LCN2* reverse: GCATACATCTTTTGCGGGTCT.

### Cell migration and cell invasion

For cell migration assay, GFP expressing tumor cells were counted, then mixed with sorted neutrophils (tumor cell: neutrophils = 1:20) and seeded in dish with silicone insert (ibidi, Munich, Germany, #81176). The silicone insert was removed when the cells grew to a monolayer covering the bottom surface. The images were photographed and measured using a fluorescence microscope at 0, 24 hours.

For cell invasion assay, 1:8 dilution of matrix gel (Corning Matrigel, #356234) was added in the upper of the transwell chamber, and placed in 37 ℃ waiting for the matrix gel to solidify. Add 100 μL PBS to the upper chamber and discard after 2 hours. Tumor cells were co-cultured with neutrophils (tumor cell: neutrophils = 1:20) for 24 hours and were separated by MACS, then sorted tumor cells were counted and 60 μL cell suspension was seeded in the upper chamber. 100 μL complete medium was added to the lower chamber. the chamber was removed after 48 hours, upper chamber liquid was aspirated and the chamber was fixed in 4% paraformaldehyde for 10 minutes. The upper chamber matrix gel was carefully wiped out and stained in crystal violet staining solution for 5 minutes, observed and photographed under the microscope.

### Public database obtain

The data showed in the manuscript was downloaded from TCGA dataset, SEER dataset, TIMER database (https://cistrome.shinyapps.io/timer/) and STRING database (https://string-db.org/). Briefly, data of breast cancer patients was obtained and divided into different molecular subtype. Survival analysis was performed with K-M plotter database (https://kmplot.com/analysis/) and SPSS statistical software (Version 25.0). Other differential factor expression was analyzed using package (version 3.2.5) and Graphpad Prism (Version 9.0)

### Statistics

Statistical analysis was performed using Graphpad Prism (Version 9.0) software. Statistical significance (*p <0.05, **p <0.01, ***p <0.001 and ****p <0.0001) between the means of a minimum of three groups was determined using unpaired two-tailed Student’s t test, two-way ANOVA. Results are expressed as the mean value ± SD. All results are representative of at least three independent experiments.

## Results

### Increased numbers of tumor-associated neutrophils in TNBC are associated with poor prognosis

**Fig. 1:**
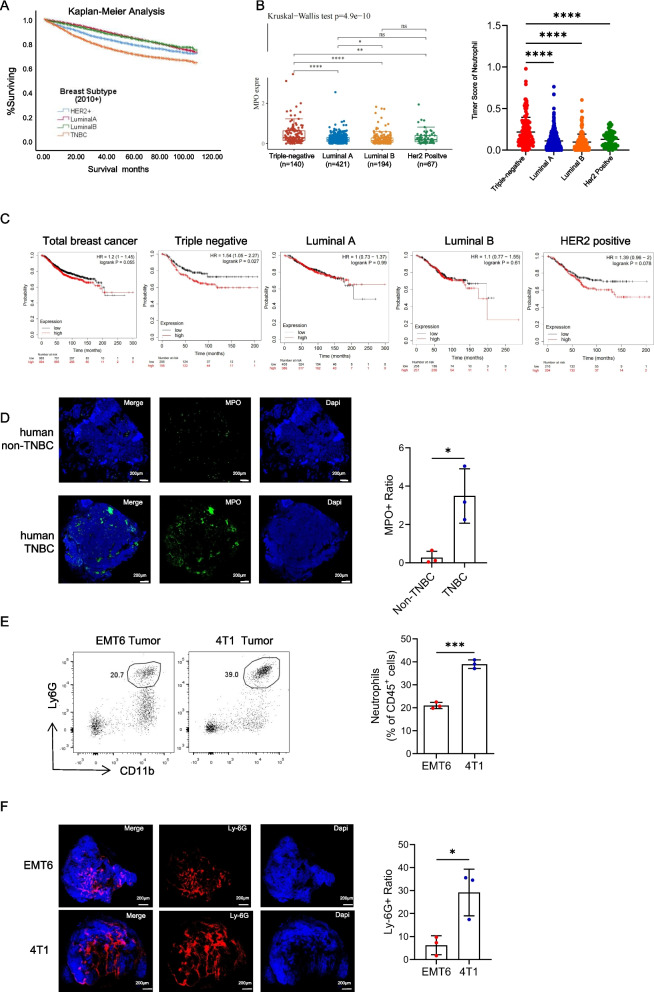
Increased numbers of tumor-associated neutrophils in triple-negative breast cancer are associated with poor prognosis. A. Prognostic analysis of different molecular types of breast cancer in SEER database. **B** Quantitative analysis of MPO expression in primary breast cancer of different molecular subtypes in TCGA database. **C** Analysis of the correlations between CEACAM8 (CD66b) expression in primary tumors with overall survival (OS) in patients with breast cancer from the TCGA database. **D** Images of immunofluorescence staining of tumor infiltrating neutrophil in the human non-triple negative breast cancer (non-TNBC) and triple negative breast cancer (TNBC). Green, MPO. Blue, DAPI. Scale bar, 200 μm. **E** Flow cytometry analysis and quantification of tumor infiltrating neutrophils (CD45^+^CD11b^+^Ly-6G^+^) from the EMT6 non-triple negative breast cancer model and 4T1 triple negative breast cancer model. **F** Images of immunofluorescence staining of tumor infiltrating neutrophil in the EMT6 model and 4T1 model. Red, Ly-6G. Blue, DAPI. Scale bar, 200 μm. Data are presented as the means ± SD from one representative experiment. Similar results were obtained from three independent experiments, unless indicated otherwise. Statistical analysis was performed by Kaplan-Meier analysis (A, C), Kruskal-Wallis test (B) and two-tailed unpaired Student's t test (D, E). ns, not significant, *p˂0.05, **p˂0.01, ***p˂0.001, and ****p˂0.0001

TNBC is the most aggressive molecular subtype of breast cancer. Our analysis of the SEER database further supported the notion that TNBC patients exhibit the poorest prognosis, followed by patients with HER2-positive breast cancer (Fig. 1A). Known for its heightened immunogenicity, TNBC is characterized by increased somatic mutations and immune cell infiltration. In the TCGA database, TNBC exhibited the highest expression of MPO (a neutrophil-specific marker) (Fig. 1B). This led us to investigate the relationship between neutrophil counts and patient prognosis. Although neutrophil infiltration lacked prognostic significance in breast cancer overall, our analysis of the TCGA database revealed a starkly different scenario in TNBC, where increased neutrophil levels were strongly associated with worse prognoses across different subtypes (Fig. 1C). Subsequent validation through fluorescence staining of neutrophils demonstrated a more pronounced infiltration of neutrophils in TNBC (Figure 1D). Further examination of mouse tumor tissues revealed significantly more neutrophils in the TNBC (4T1) model than in the non-TNBC (EMT6) model (Fig. 1E and F). Based on these findings, we postulate that neutrophils may play a unique role in TNBC, contributing significantly to its poor prognosis.

### TNBC cells adhere closely to neutrophils, facilitating alterations in their function

**Fig. 2:**
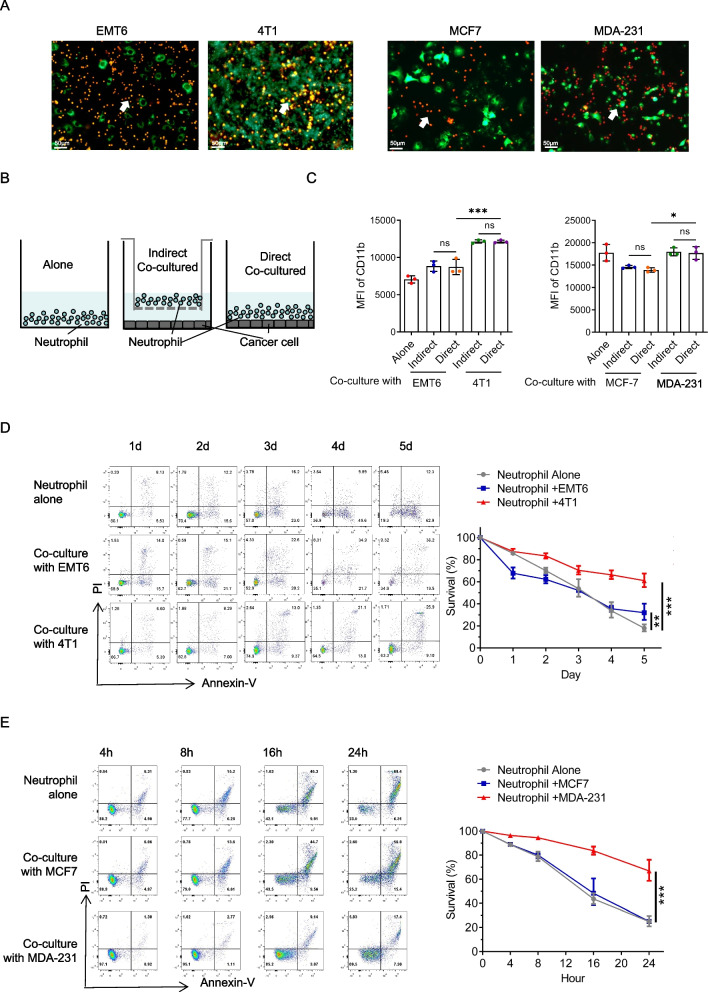
Triple-negative breast cancer cells adhere closely to neutrophils, facilitating alterations in their function. **A** Alive co-cultured imaging of neutrophils and tumor cells. Red, Dil-labled neutrophil. Green, GFP. Scale bar, 50 μm. Tumor cells: neutrophils = 1:20. Mice neutrophils were isolated from the bone marrow (BM) of naive mice and human neutrophils were isolated from peripheral blood of patients with benign breast tumor. **B** Schematic diagram of different co-culture system *in vitro*. **C** Flow cytometry analysis of CD11b expression in neutrophils with different interventions for 24 hours. **D**-**E** Flow cytometry analysis and quantification of survival dynamics on mice (**D**) and human (**E**) neutrophils in different conditioned tumor cell cultured supernatants. Data are presented as the means ± SD from one representative experiment. Similar results were obtained from three independent experiments, unless indicated otherwise. Statistical analysis was performed by one-way ANOVA (**C**) and Kaplan-Meier analysis (**D**, **E**). ns, not significant, *p˂0.05, **p˂0.01, and ***p˂0.001

To investigate the interaction between neutrophils and tumor cells, we isolated mature neutrophils from the bone marrow of naive mice and from the peripheral blood of patients with benign breast tumors. These neutrophils were then co-cultured with different tumor cells of the corresponding species. Intriguingly, we observed specific adherence of neutrophils to TNBC cells (4T1 and MDA-MB-231), while non-TNBC cells (EMT6 and MCF7) did not display such binding tendencies (Fig. 2A). This adhesive phenomenon potentially clarifies the heightened presence of neutrophils in TNBC.

To further explore this possibility, we examined the expression of CD11b, a crucial adhesion molecule on neutrophils, during direct and indirect co-culture with tumor cells. Interestingly, we discovered that CD11b expression on neutrophils was elevated in TNBC cells during both direct and indirect co-culture, whereas no such increase was observed in non-TNBC cells (Fig. 2B and C). This upregulation could be mediated by cytokine releasing from tumor cells. Moreover, coculturing neutrophils with TNBC cell supernatant significantly prolonged neutrophil survival, whereas non-TNBC cell supernatant did not have a similar effect (Fig. 2D and E and Figure S1A). Consequently, we postulate that TNBC cells can promote neutrophil adhesion, prolong neutrophil survival, and increase neutrophil CD11b expression.

### GM-CSF has emerged as a pivotal factor driving the longevity and enhanced adhesion capacity of neutrophils

**Fig. 3:**
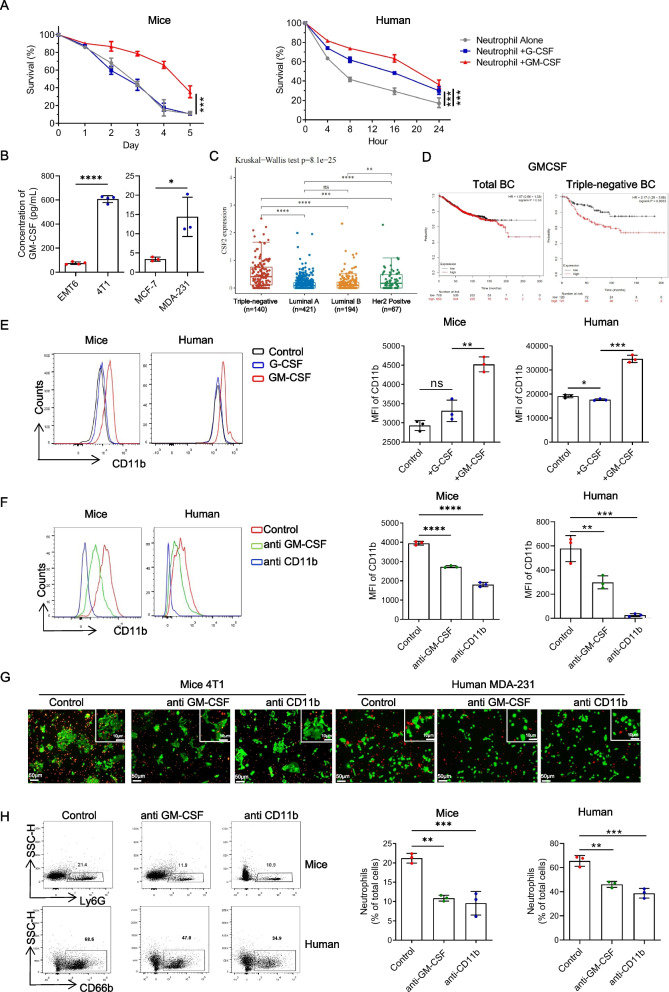
GM-CSF emerges as a pivotal factor driving the longevity and enhanced adhesion capacity of neutrophils. **A** Flow cytometry analysis and quantification of survival dynamics in mice and human neutrophils treated with G-CSF (50 ng/mL) or GM-CSF (50 ng/mL). Mice neutrophils were isolated from the bone marrow (BM) of naive mice and human neutrophils were isolated from peripheral blood of patients with benign breast tumor. **B** Concentration of GM-CSF in different tumor cell cultured supernatants. C Quantitative analysis of *CSF2 (GM-CSF)* expression in primary breast cancer of different molecular subtypes in TCGA database. **D** Analysis of the correlations between *CSF2 (GM-CSF)* expression in primary tumors with overall survival (OS) in patients with total breast cancer and triple negative breast cancer from the TCGA database. **E** Flow cytometry analysis and quantification of CD11b in neutrophils treated with G-CSF (50 ng/mL) or GM-CSF (50 ng/mL) for 24 hours. **F** Flow cytometry analysis and quantification of CD11b in neutrophils treated with anti GM-CSF mAbs (10 ng/mL) or anti CD11b mAbs (10 ng/mL) for 24 hours. **G** Live co-cultured imaging of tumor cells and neutrophils after 24 hours treatment after treatment with anti-CD11b mAbs (10 ng/mL) or anti-GM-CSF mAbs (10 ng/mL) for 24 hours. Red, Dil-labled neutrophil. Green, GFP. Scale bar, 50 μm. Tumor cells: neutrophils = 1:20. **H** Flow cytometry analysis and quantification of neutrophils in the co-cultured systems from Figure 3G. Data are presented as the means ± SD from one representative experiment. Similar results were obtained from three independent experiments, unless indicated otherwise. Statistical analysis was performed by Kaplan-Meier analysis (**A**), two-tailed unpaired Student's t test (**B**), and one-way ANOVA (**E**, **F**, **H**). ns, not significant, *p˂0.05, **p˂0.01, ***p˂0.001, and ****p˂0.0001

Granulocyte colony-stimulating factor (G-CSF) and granulocyte-macrophage colony-stimulating factor (GM-CSF) play crucial roles in neutrophil development and survival [25]. We observed greater G-CSF and GM-CSF expression levels in TNBC cells than in non-TNBC cells, with GM-CSF displaying a particularly pronounced difference (Figure S1B). We induced neutrophils *in vitro* using G-CSF and GM-CSF separately to unravel their effects. Intriguingly, GM-CSF, but not G-CSF, significantly prolonged the lifespan of mature neutrophils (Fig. 3A and Figure S1C). Furthermore, analysis of tumor cell culture supernatants revealed elevated levels of GM-CSF secreted by TNBC cells, underscoring its potential significance in TNBC (Fig. 3B). Moreover, scrutiny of the TCGA database revealed TNBC as the molecular subtype with the highest GM-CSF expression, while no significant difference among breast cancer subtypes was observed for G-CSF (Fig. 3C and Figure S1D). Prognostic analysis indicated that GM-CSF exhibited a significant association with a poor prognosis in only TNBC patients, without prognostic relevance in total breast cancer patients or patients with other molecular subtypes of breast cancer (Fig. 3D and Figure S1E). Additionally, the expression level of G-CSF lacked prognostic significance in all breast cancer molecular subtypes (Figure S1F). These findings highlight the critical role of GM-CSF, rather than G-CSF, in TNBC. Subsequent induction experiments demonstrated that GM-CSF, not G-CSF, upregulated CD11b expression in neutrophils (Fig. 3E). Consequently, we performed antagonism experiments targeting GM-CSF and CD11b, which revealed a reduction in the impact of tumor cells on CD11b upregulation in neutrophils within the co-culture system (Figure 3F). To validate these outcomes, we treated the co-culture system with anti-GM-CSF and anti-CD11b and observed a noteworthy decrease in neutrophil adhesion to TNBC cells (Fig. 3G and H). Thus, we propose that GM-CSF secretion in TNBC promotes the upregulation of CD11b expression in neutrophils, contributing to increased neutrophil adhesion.

### Neutrophil CD11b directly binds to the tumor cell ICAM1, facilitating cell adhesion

**Fig. 4:**
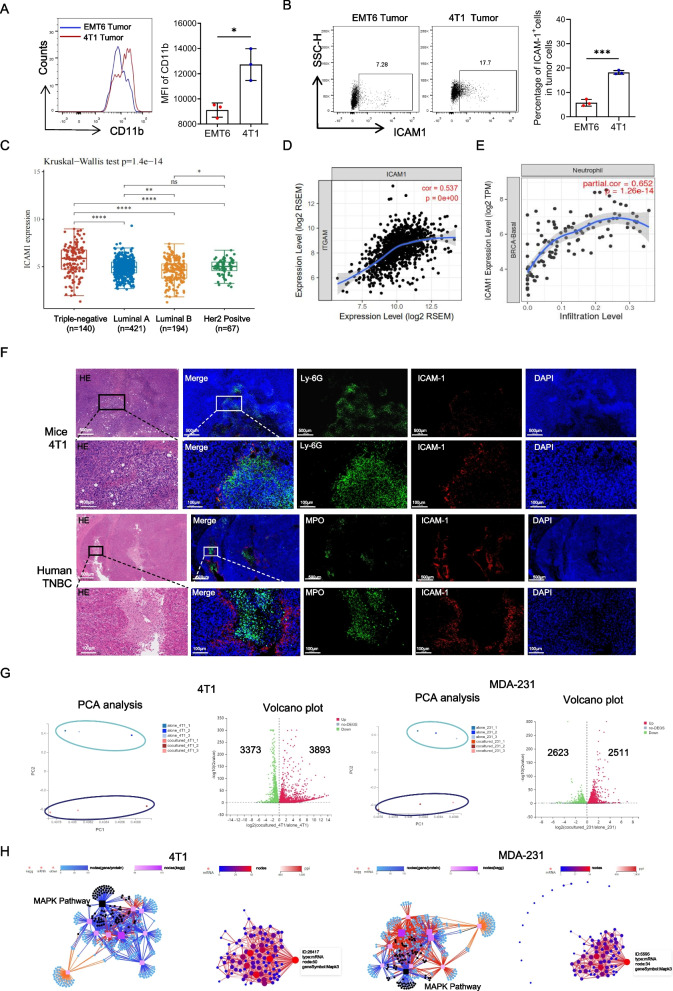
Neutrophil CD11b directly binds to the tumor cell ICAM1, facilitating cell adhesion. **A** Flow analysis of CD11b in tumor infiltrating neutrophils from 4T1 or EMT6 tumor bearing mice at 2 weeks. **B** Flow analysis of ICAM1 in tumor cells from 4T1 or EMT6 tumor bearing mice at 2 weeks. **C** Quantitative analysis of ICAM1 expression in primary breast cancer of different molecular subtypes in TCGA database. **D** Pearson analysis of the correlations between ITGAM (CD11b) expression with ICAM1 expression in primary tumors with breast cancer from the TCGA database. **E** Pearson analysis of the correlations between ICAM1 expression with neutrophil infiltration in primary tumors with TNBC from the TCGA database. **F** Representative H&E and paired immunofluorescence staining images of neutrophils and ICAM1 in tumor from 4-week tumor bearing mice and TNBC patient. Red, ICAM1. Green, Ly6G (neutrophil in mice). MPO (neutrophil in human). Blue, DAPI. Scale bar, 500 μm and 100 μm respectively. **G** Principal component analysis (PCA) (left panel) and Volcano Plot (right panel) of RNA-seq of the tumor cell co-cultured with or without neutrophils. Each dot of Volcano Plot representing a gene and genes significantly upregulated in red and downregulated in green. **H** KEGG pathway analysis (left) and protein network analysis (right) of RNA-seq of differential gene in tumor cell co-cultured with or without neutrophils. Data are presented as the means ± SD from one representative experiment. Similar results were obtained from three independent experiments, unless indicated otherwise. Statistical analysis was performed by two-tailed unpaired Student's t test (**A**, **B**), Kruskal-wallis test (**C**). ns, not significant, *p˂0.05, **p˂0.01, ***p˂0.001, and ****p˂0.0001

ICAM1, also known as CD54, acts as a ligand for CD11b and plays a pivotal role in mediating cell adhesion and initiating intracellular signal transduction by interacting with various adaptor proteins [6]. Elevated ICAM1 expression has been consistently associated with a poor prognosis across diverse tumor types [26–28]. In our investigation, we examined mouse breast cancer tissue and observed increased CD11b expression of neutrophil in TNBC compared to that in non-TNBC (Fig. 4A). Correspondingly, we also found that ICAM1 expression was significantly elevated in TNBC in contrast to non-TNBC (Fig. 4B). Analysis of the TCGA database revealed greater ICAM1 expression in breast cancer tissues than in normal breast tissue, with triple-negative breast cancer exhibiting the highest ICAM1 expression among all breast cancer subtypes (Fig. 4C and Figure S2A). Additionally, we discovered a significant positive correlation between ICAM1 expression and the expression of ITGAM (CD66b, a specific neutrophil gene) (Fig. 4D). Moreover, correlation analysis between ICAM1 and various tumor-infiltrating immune cells in different molecular subtypes of breast cancer demonstrated the strongest association between ICAM1 expression and neutrophil infiltration in TNBC (Fig. 4E and Figure S2B).

To validate the relationship between neutrophils and ICAM1 in tumor cells, we conducted immunofluorescence staining and immunohistochemistry on TNBC tissue sections from 4T1 tumor-bearing mice and breast cancer patients, revealing a clear spatial interaction between tumor ICAM1 and neutrophils (Fig. 4F and Figure S2C). Furthermore, in a co-culture system of tumor cells and neutrophils, antagonizing ICAM1 resulted in a significant reduction in the number of neutrophils adhering to the tumor cells (Figure S2D).

To investigate the impact of neutrophil–tumor cell interactions on tumors, we co-cultured neutrophils directly with TNBC cells and performed transcriptome sequencing on the sorted tumor cells (Figure S3A). Sequencing revealed significant transcriptional profile alterations in tumor cells pre- and post-coculture (Fig. 4G). KEGG pathway classification of the altered genes highlighted notable modifications in cell signal transduction, particularly in the MAPK signaling pathway, in both mouse 4T1 and human breast cancer MDA-MB-231 cells (S3B and S3C). Subsequently, we conducted signaling pathway clustering on the altered genes enriched in the MAPK pathway and identified MAPK3 as a key regulator of the tumor cell changes (Fig. 4H). To explore whether neutrophils, via their interaction with ICAM1 on tumor cells, influence intracellular signaling pathways and tumor cell malignancy, correlation analysis was conducted between multiple candidate genes affected by the MAPK pathway and ICAM1. We identified four significant downstream genes, including MMP9, STAT3, MUC1, and LCN2 (Figure S4A and S4B).

### Neutrophils enhance tumor invasion by acting on the tumor cell ICAM1 to promote the activation of the MAPK pathway in tumor cells

**Fig. 5:**
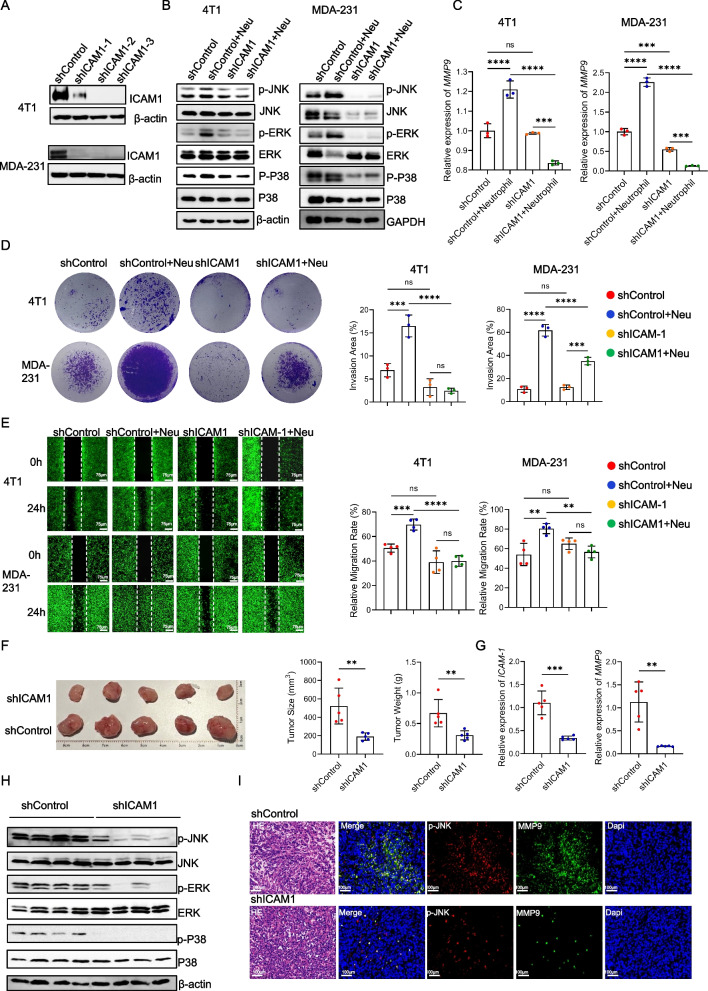
Neutrophils enhance tumor invasion by acting on the tumor cell ICAM1 to promote the activation of the MAPK pathway in tumor cells. **A** Verification of the level of the ICAM1 protein in different tumor cell lines. *shControl*, tumor cells transfected with vector lentivirus. *shICAM1-1*, *shICAM1-2*, *shICAM1-3*, tumor cells transfected with shICAM1 lentivirus of different target regions. **B** Levels of the MAPK pathway protein in different tumor cell lines co-cultured with or without neutrophils. **C** Analysis of the mRNA expression of *MMP9* in different tumor cell lines co-cultured with or without neutrophils. **D** Invasion assay images and quantification of different tumor cell lines co-cultured with or without neutrophils. **E** Migration capability images and quantification of different tumor cell lines co-cultured with or without neutrophils. **F** Image (left panel) and tumor weights (right panel) of primary tumors from 4-week tumor bearing mice inoculated with *shControl*- or *shICAM1*-4T1 cells. **G** Analysis of the mRNA expression of *ICAM1* and *MMP9* in primary tumors from 4-week tumor bearing mice inoculated with *shControl*- or *shICAM1*-4T1 cells. **H** Levels of the MAPK pathway protein in primary tumors from 4-week tumor bearing mice inoculated with *shControl*- or *shICAM1*-4T1 cells. **I** Representative H&E and paired immunofluorescence staining images of p-JNK and MMP9 in tumor from 4-week tumor bearing mice inoculated with *shControl*- or *shICAM1*-4T1 cells. Red, p-JNK. Green, MMP9. Blue, DAPI. Scale bar, 100 μm. Data are presented as the means ± SD from one representative experiment. Similar results were obtained from three independent experiments, unless indicated otherwise. Statistical analysis was performed by two-tailed unpaired Student's t test (F, G) and one-way ANOVA (C, D, E). ns, not significant, *p˂0.05, ***p˂0.001, and ****p˂0.0001

To investigate the role of tumor cell ICAM1, we established stable *shICAM1* cell lines, specifically the 4T1-*shICAM1*-2 and MDA-MB-231-*shICAM1*-2 cell lines, for subsequent experiments (Fig. 5A, Figure S5A and S5B). Coculturing tumor cells with varying levels of ICAM1 expression alongside neutrophils enabled us to examine the MAPK signaling pathway in tumor cells. We observed that neutrophils significantly upregulated the MAPK pathway in tumor cells, with p-JNK and p-ERK exhibiting the most prominent changes. Notably, knocking down ICAM1 counteracted the impact of neutrophils on the MAPK pathway (Fig. 5B and Figure S5C). Furthermore, we evaluated the expression of potential downstream genes and discovered that ICAM1 knockdown abolished *MMP9* and *STAT3* expression (Fig. 5C and S5D). Functional assessments of cell migration and invasion demonstrated that neutrophils substantially increased tumor cell invasion and migration, while ICAM1 knockdown mitigated the promoting effect of neutrophils on tumor cells (Fig. 5D and E).

**Fig. 6:**
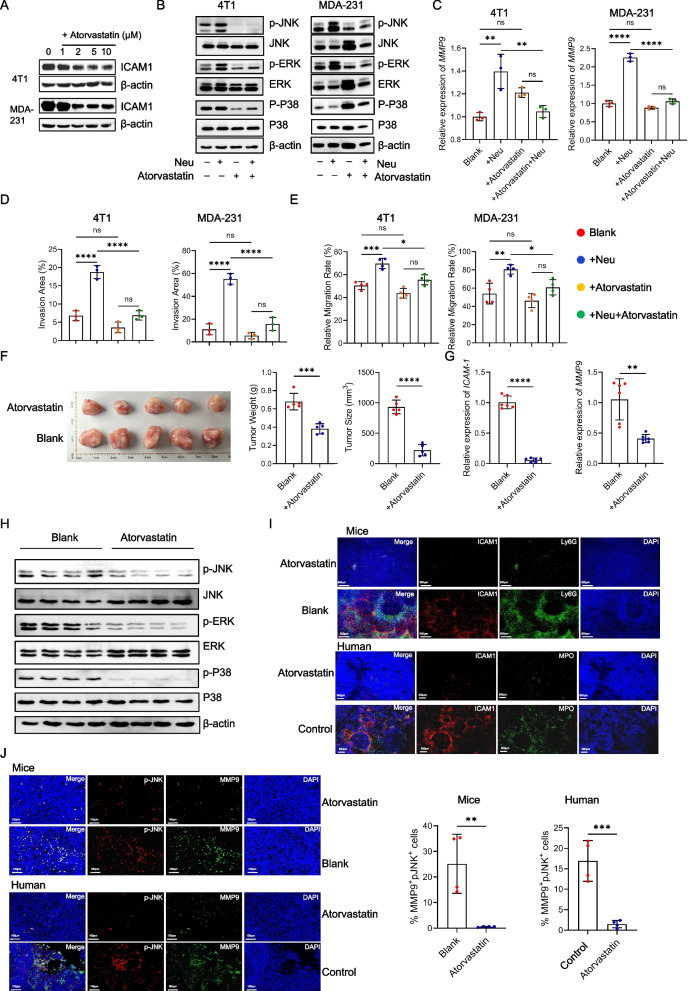
Atorvastatin can inhibit ICAM1 in tumor cells and reduce the malignant characteristics of TNBC. **A** Levels of the ICAM1 protein on tumor cell lines treated with atorvastatin for 24 hours *in vitro*. **B** Levels of the MAPK pathway protein in tumor cell lines co-cultured with or without neutrophils in the presence or absence of atorvastatin (4T1 10 μM, MDA-MB-231 5 μM) for 24 hours *in vitro*. **C** Analysis of the mRNA expression of *MMP9* in tumor cell lines co-cultured with or without neutrophils in the presence or absence of atorvastatin for 24 hours *in vitro*. **D** Invasion assay images and quantification of tumor cell co-cultured with or without neutrophils in the presence or absence of atorvastatin for 24 hours *in vitro*. **E** Migration capability images and quantification of tumor cell lines co-cultured with or without neutrophils in the presence or absence of atorvastatin for 24 hours *in vitro*. **F** Image (left panel) and tumor weights (right panel) of primary tumors from 4-week tumor bearing mice inoculated with 4T1 cells after the intragastric administration of atorvastatin or PBS. **G** Analysis of the mRNA expression of *ICAM1* and *MMP9* in primary tumors from 4-week tumor bearing mice inoculated with 4T1 cells after the intragastric administration of atorvastatin or PBS. **H** Levels of the MAPK pathway protein in primary tumors from 4-week tumor bearing mice inoculated with 4T1 cells after the intragastric administration of atorvastatin or PBS. **I** Representative immunofluorescence staining images of neutrophils and ICAM1 in tumor from 4-week tumor bearing mice and triple negative patient treated with or without atorvastatin. Red, ICAM1. Green, Ly6G (neutrophil in mice). MPO (neutrophil in human). Blue, DAPI. Scale bar, 500 μm. **J** Representative immunofluorescence staining images of p-JNK and MMP9 in tumor from 4-week tumor bearing mice and triple negative patient treated with or without atorvastatin. Red, p-JNK. Green, MMP9. Blue, DAPI. Scale bar, 100 μm. Data are presented as the means ± SD from one representative experiment. Similar results were obtained from three independent experiments, unless indicated otherwise. Statistical analysis was performed by two-tailed unpaired Student's t test (F, G, J) and one-way ANOVA (C, D, E). ns, not significant, *p˂0.05, **p˂0.01, ***p˂0.001, and ****p˂0.0001

To further elucidate the relationship between tumor ICAM1 and MAPK, we established a breast cancer mouse model using *shControl* and *shICAM1* cells. We observed that the tumors in the *shICAM1* group were significantly smaller than those in the *shControl* group (Fig. 5F). Additionally, ICAM1 and MMP9 expression was significantly reduced in *shICAM1* tumors (Fig. 5G and Figure S6A). We also examined neutrophil CD11b expression and found varying degrees of decline in the *shICAM1* group (Figure S6C). The expression of the MAPK pathway was significantly downregulated in *shICAM1* tumor tissues (Fig. 5H and Figure S6D). Immunofluorescence and immunohistochemistry analyses revealed significant colocalization between p-JNK, a representative MAPK pathway protein, and MMP9 within tumor tissues. Remarkably, the *shICAM1* group exhibited significantly reduced p-JNK and MMP9 expression in tumor cells (Fig. 6I and Figure S6F). These findings underscore the role of neutrophils in interacting with ICAM1 on tumor cells, thereby triggering MAPK signaling activation and subsequently fostering tumor cell invasion and migration.

### Atorvastatin can inhibit ICAM1 in tumor cells and reduce the malignant characteristics of TNBC

Atorvastatin, a widely recognized cholesterol-lowering medication and HMG-CoA reductase inhibitor, has been reported to decrease ICAM1 expression and secretion [29]. We treated TNBC cells with atorvastatin and observed a significant reduction in ICAM1 expression (Fig. 6A and Figure S7A). Subsequent administration of atorvastatin in the co-culture system of neutrophils and tumor cells effectively inhibited the activation of the MAPK pathway initiated by neutrophils in tumor cells (Fig. 6B and Figure S7B). Analysis of downstream target genes revealed significant inhibition of *MMP9* and *STAT3* (Fig. 6C and Figure S7C). Moreover, atorvastatin intervention significantly counteracted the ability of neutrophils to promote tumor cell invasion and migration (Fig. 6D and E, Figure S7D and S7E). To assess its effects *in vivo*, we administered atorvastatin via intragastric administration in a 4T1 mouse breast cancer model. Although the reduction in tumor size was partial compared to the change observed in *shICAM1* tumor tissue, atorvastatin treatment yielded promising results (Fig. 6F). Additionally, tumor tissue analysis revealed a significant decrease in *ICAM1, MMP9* and *MUC1* expression following atorvastatin treatment (Fig. 6G and Figure S8A), along with inhibited MAPK pathway activation (Fig. 6H and Figure S8B). We examined neutrophil expression in tumor tissues and found that the number of neutrophils was decreased, as was the expression of CD11b (Figure S8C and S8D).

To further validate these findings in clinical settings, we performed fluorescence staining on tumor specimens from patients with clinical triple-negative breast cancer who had received long-term atorvastatin treatment for hyperlipidemia, as well as from those who had never received long-term statin therapy. The results revealed significant direct contact between neutrophils and ICAM1 in untreated tumor tissues. However, following statin treatment, there was a significant decrease in the expression ICAM1 and the presence of neutrophils in tumor tissues, consistent with the findings observed in the TNBC mouse model (Fig. 6I). Additionally, we performed fluorescence staining for p-JNK and MMP9. The fluorescence results demonstrated significant colocalization of p-JNK and MMP9 in triple-negative breast cancer, with significant inhibition of p-JNK and MMP9 expression after atorvastatin treatment (Fig. 6J). These findings suggest that atorvastatin holds promise as a clinical drug for inhibiting ICAM1 expression in triple-negative breast cancer. Moreover, this approach has the potential to diminish neutrophil-mediated activation of the MAPK pathway in tumor cells and has significant novel clinical application value.

## Discussion

In this article, we provide insights into the interaction between neutrophils and tumor cells in triple-negative breast cancer, highlighting their contribution to tumor progression. We demonstrated that tumor cells exhibit elevated GM-CSF expression, which facilitates the recruitment and persistence of neutrophils in the tumor microenvironment. Consequently, neutrophils upregulate the expression of CD11b. The binding of CD11b to ICAM1 on tumor cells triggers activation of the MAPK pathway within tumor cells, leading to the upregulation of MMP9 expression. Ultimately, this cascade of events promotes tumor cell migration and invasion.

During development, cell lineages undergo multiple decision points where they differentiate into various mature cells under the influence of cytokines. The fate of myeloid progenitor cells is determined by the regulation of GM-CSF, G-CSF, and M-CSF, which ultimately dictate their destiny [25]. Agranulocytosis following chemotherapy is a significant indication for the use of GM-CSF in cancer patients due to its mobilization effect on the bone marrow.

However, a substantial body of literature has demonstrated that GM-CSF acts as a ‘double-edged sword’ in cancer. The production of GM-CSF by tumor cells is associated with a favorable clinical prognosis in colorectal cancer patients [30]. GM-CSF can activate antitumor T cells [31], inhibit tumor angiogenesis [32], and even directly impede tumor growth [33], suggesting that GM-CSF, as a chemotherapy adjuvant, may contribute to this antitumor effect. In contrast, the upregulation of GM-CSF has been linked to a poor prognosis in bladder cancer and head and neck squamous cell carcinoma [34, 35]. GM-CSF plays a crucial role in promoting the generation of tumor-associated macrophages and myeloid-derived suppressor cells, which represents its most significant biological mechanism. Numerous studies have demonstrated that inhibiting the differentiation and maturation of myeloid progenitor cells is vital for maintaining a suppressive immune microenvironment [36, 37]. However, the biological function of GM-CSF in mature, terminally differentiated myeloid cells remains unclear.

Using sorted mature neutrophils from the peripheral blood of patients and mouse bone marrow-derived mature neutrophils, our findings indicate that GM-CSF, but not G-CSF, consistently enhances neutrophil survival and upregulates CD11b expression *in vitro*. CD11b, in addition to being a myeloid cell marker, serves as a critical cell surface adhesion molecule [38]. Blocking CD11b and GM-CSF in co-culture systems effectively reduces the adhesion between neutrophils and tumor cells, further supporting the impact of GM-CSF on neutrophil adhesion. The prognostic significance of GM-CSF in TNBC is not only associated with its highest expression in TNBC but also linked to the unique interaction between neutrophils and tumor cells in TNBC.

Previous studies have highlighted the significant role of neutrophils in breast cancer progression, particularly as crucial components of the circulatory system with a well-established function in mediating tumor metastasis. Szczerba et al. demonstrated that circulating neutrophils can form cell clusters with circulating tumor cells, providing protection against the autoimmune system and facilitating their colonization of distant organs [39]. Furthermore, several studies have indicated that neutrophils in distant organs contribute to the establishment of an immunosuppressive microenvironment known as the premetastatic niche, which supports tumor cell residence and growth [22, 40–42].

However, unlike the high content of tumor-infiltrating lymphocytes or macrophages, the proportion of neutrophils within the immune cell population in the primary breast cancer tumor microenvironment is not large [43]. Consequently, the role of tumor-infiltrating neutrophils remains incompletely understood and contentious within the field [44]. Studies have revealed the plasticity of tumor-associated neutrophils (TANs), which are influenced by the local microenvironment [45–47], leading to their polarization into either the antitumor N1 type or protumor N2 type [48].

The current understanding of TANs primarily focuses on their role in promoting tumor growth by creating an immunosuppressive microenvironment, recruiting macrophages and Treg cells, and inhibiting CTL infiltration and cytotoxic function [49–53]. However, the direct impact of TANs on tumor cells remains underexplored. Some researchers have shown that neutrophils can enhance tumor proliferation, migration, invasion, and treatment resistance [54, 55], but the underlying mechanisms are still unclear. Notably, neutrophils have prognostic implications in TNBC patients, emphasizing the need for further investigation in this context.

Our analysis of TNBC patient and mouse tumor sections revealed that neutrophils primarily localized around tumor cells exhibiting high ICAM1 expression. *In vitro* experiments demonstrated the specific adhesion of neutrophils to TNBC cells. These findings suggest a direct interaction between neutrophils and TNBC cells, with CD11b inhibition significantly attenuating this adhesion. Thus, CD11b-mediated direct contact may represent a crucial mechanism by which neutrophils contribute to the progression of TNBC.

ICAM1, acting as the receptor for CD11b, is typically involved in facilitating intercellular adhesion. For instance, it promotes adhesion between tumor cells and endothelial cells, facilitating tumor cell extravasation and metastasis, as well as adhesion between leukocytes and endothelial cells, supporting local leukocyte residence [56, 57]. Both CD11a and CD11b serve as ligands for ICAM1 in lymphocytes, endothelial cells, and other mesenchymal cells, mediating cell motility and adhesion. Through correlation analysis, we discovered a strong association between ICAM1 and neutrophils in the tumor microenvironment, particularly in TNBC, suggesting a potential interaction between ICAM1 and neutrophils in TNBC. Previous studies have highlighted the elevated expression of ICAM1 in TNBC, making it a promising therapeutic target [8]. Consequently, the development of antibody-conjugated drugs (ADCs) targeting ICAM1 has shown efficacy in treating advanced TNBC in animal models [58]. Current research is focused on elucidating the mechanisms that induce ICAM1 expression, including the JAK-STAT pathway and the TNF-related pathway [59].

Nevertheless, few studies have investigated the downstream signaling pathways activated by ICAM1. Among the limited mechanistic investigations, ICAM1 has been shown to engage with *SRC*, leading to increased *SRC* activity. Consequently, ICAM1 may potentiate *SRC* signaling, thereby promoting the malignant potential of cancer [60].

In our study, we conducted transcriptome sequencing of tumor cells before and after co-culture with neutrophils. By analyzing the interaction between differentially expressed genes and ICAM1, we observed the enrichment of these altered genes in the MAPK signaling pathway. Furthermore, the CD11b-ICAM1-MAPK pathway was elucidated through the interaction of *shICAM1* with neutrophils. Additionally, we explored the target genes within the MAPK pathway. Intriguingly, *in vitro* results demonstrated that inhibiting ICAM1 could counteract the activation of the MAPK pathway by neutrophils, subsequently reducing the upregulation of MMP9 and STAT3. However, the *in vivo* findings did not confirm an influence of the ICAM1-MAPK pathway on STAT3. *In vivo* experiments revealed co-expression of ICAM1 and MMP9 on tumor cells. Inhibiting ICAM1 significantly suppressed MAPK pathway activation, decreased MMP9 expression, and resulted in notable tumor shrinkage. Moreover, we explored relevant target genes within the MAPK pathway.

The role of ICAM1 extends beyond inflammation and cancer, as it has been extensively investigated in atherosclerosis [61]. Atorvastatin, a competitive inhibitor of hydroxymethylglutaryl-coenzyme A (HMG-CoA) reductase, acts by inhibiting an enzyme involved in cholesterol synthesis. Consequently, it reduces cholesterol levels in the bloodstream, and is commonly used to treat patients with hyperlipidemia. Notably, research has demonstrated the potential antitumor effects of atorvastatin, particularly through the AKT/mTOR and caspase-3 pathways, which exhibit remarkable antitumor efficacy [62, 63]. In a phase II window-of-opportunity trial involving breast cancer patients, preoperative administration of atorvastatin led to a reduction in tumor proliferation [64]. These findings further support the notion that atorvastatin possesses anti-breast cancer properties in addition to its lipid-lowering effects [65].

## Conclusion

Our study revealed that statins effectively suppressed ICAM1 expression and counteracted the activation of the MAPK pathway downstream of tumor cells by neutrophils. *In vivo* administration of statins in mice resulted in smaller primary breast cancer lesions, albeit to a lesser extent than shICAM1 did. Furthermore, we screened newly diagnosed breast cancer patients who had been receiving atorvastatin treatment for more than 1 year. Tumor tissue staining demonstrated a significant reduction in neutrophils and ICAM1 within the tumors, accompanied by inhibited expression of the MAPK pathway and its downstream target MMP9. These findings underscore the crucial role of atorvastatin in the treatment of triple-negative breast cancer, particularly in regulating the interaction between tumor cells and neutrophils.

### Supplementary Information

Supplementary Material 1.

## Data Availability

No datasets were generated or analysed during the current study.

## Abbreviations

TNBC: Triple-negative breast cancer
Her2: human epidermal growth factor receptor 2
TILs: tumor-infiltrating lymphocytes
TAN: tumor associated neutrophils
MPO: Myeloperoxidase
G-CSF: Granulocyte colony-stimulating factor
GM-CSF: granulocyte-macrophage colony-stimulating factor
shRNA: short hairpin RNA
