# Supplementary material for: Mechanisms underlying neutrophils adhesion to triple-negative breast cancer cells via CD11b-ICAM1 in promoting breast cancer progression
Source: Cell Commun Signal. 2024 Jun 21;22:340. doi: 10.1186/s12964-024-01716-5 (PMC11191284; doi:10.1186/s12964-024-01716-5)
Supplement: Supplementary file 1 — Supplementary Material 1. [file 12964_2024_1716_MOESM1_ESM.pdf]

Mechanisms underlying neutrophils adhesion to triple-negative breast cancer cells via CD11b-ICAM1 in promoting breast cancer progression

Supplementary Materials

(Containing 8 Supplementary Figures)

Figure S1. Related to Figure 2 and 3

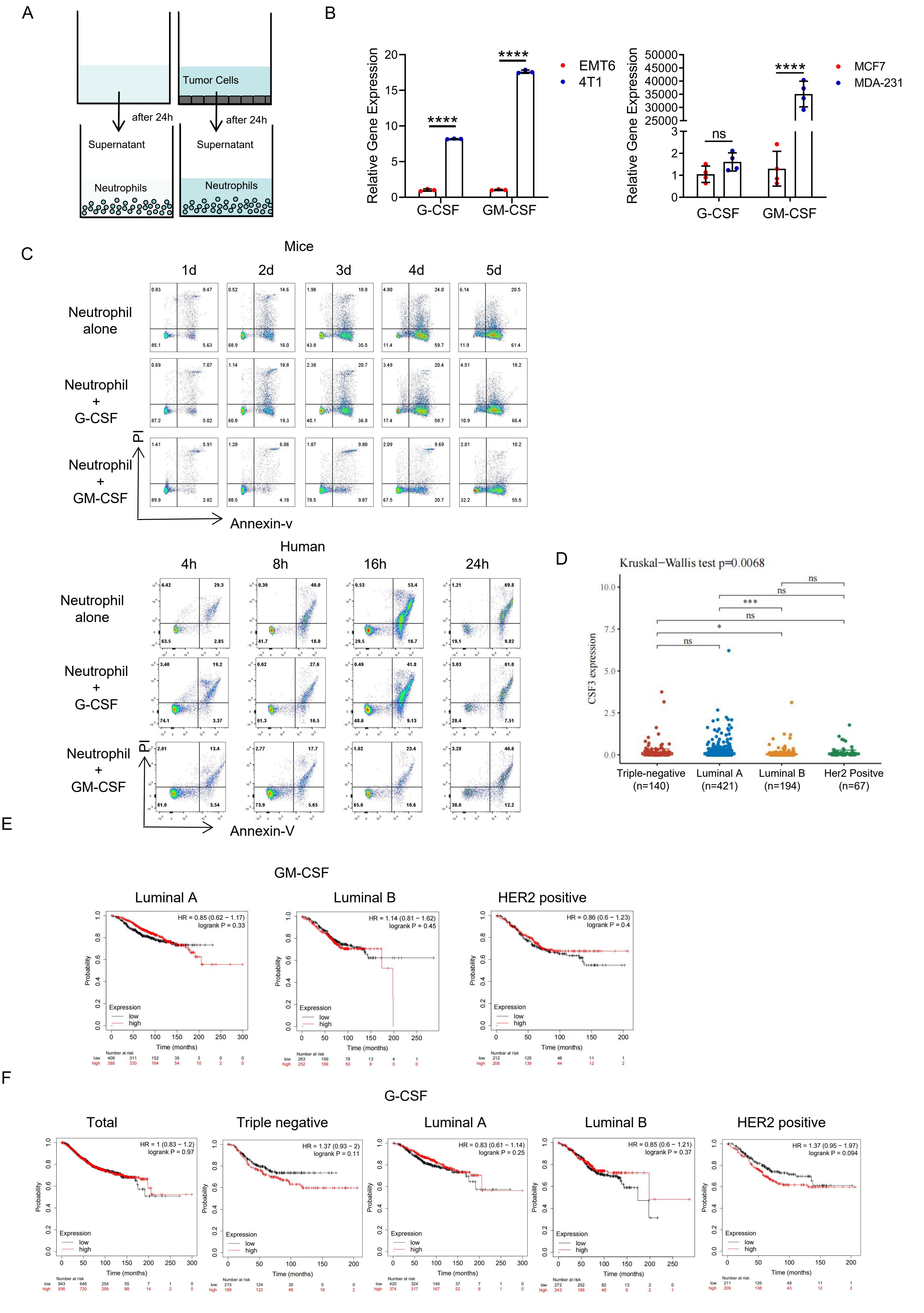

Figure S1. Related to Figure 2 and 3

(A) Schematic diagram of neutrophils conditional medium in Figure 2D and 2E *in vitro*.

(B) Expression of the *G-CSF* and *GM-CSF* mRNA in tumor cell lines.

(C) Flow cytometry analysis of survival dynamics in mice and human neutrophils treated with G-CSF (50 ng/mL) or GM-CSF (50 ng/mL) in Figure 3A.

(D) Quantitative analysis of *CSF3 (G-CSF)* expression in primary breast cancer of different molecular subtypes in TCGA database.

(E) Analysis of the correlations between *CSF2 (GM-CSF)* expression in primary tumors with overall survival (OS) in patients with Luminal A, Luminal B and Her2 positive breast cancer from the TCGA database.

(F) Analysis of the correlations between *CSF3 (G-CSF)* expression in primary tumors with OS in patients with different breast cancer from the TCGA database.

Data are presented as the means ± SD from one representative experiment. Similar results were obtained from three independent experiments, unless indicated otherwise. Statistical analysis was performed by two-tailed unpaired Student's t test (B) and Kruskal-Wallis test (D). ns, not significant, *p<0.05, **p<0.01, ***p<0.001, and ****p<0.0001.

Figure S2. Related to Figure 4

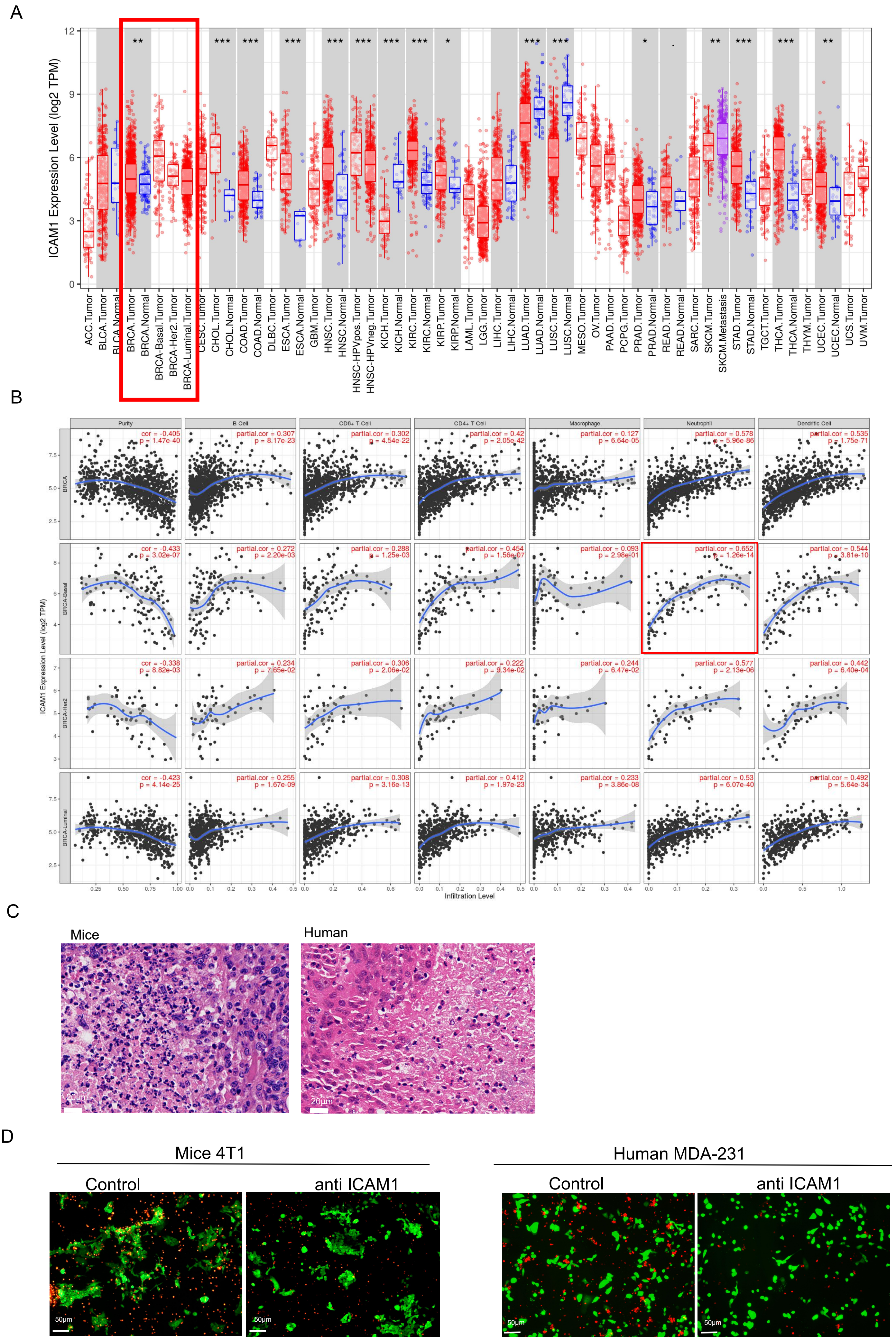

Figure S2. Related to Figure 4

(A) Quantitative analysis of ICAM1 expression in different cancers in TCGA database.

(B) Pearson analysis of the correlations between ICAM1 expression with subgroup of immune cells infiltration in primary tumors with different molecular subtypes breast cancer from the TCGA database.

(C) Representative H&E images of tumor from 4-week tumor bearing mice and TNBC patient in Figure 4F. Scale bar, 20μm.

(D) Live co-cultured imaging of tumor cells and neutrophils after 24 hours treatment after treatment with anti-ICAM1 mAbs (10 ng/mL) for 24 hours. Red, Dil-labled neutrophil. Green, GFP. Scale bar, 50 μm. Tumor cells: neutrophils = 1:20.

Data are presented as the means ± SD from one representative experiment. Similar results were obtained from three independent experiments, unless indicated otherwise. Statistical analysis was performed by one-way ANOVA (A). ns, not significant, *p<0.05, **p<0.01, and ***p<0.001.

Figure S3. Related to Figure 4

A

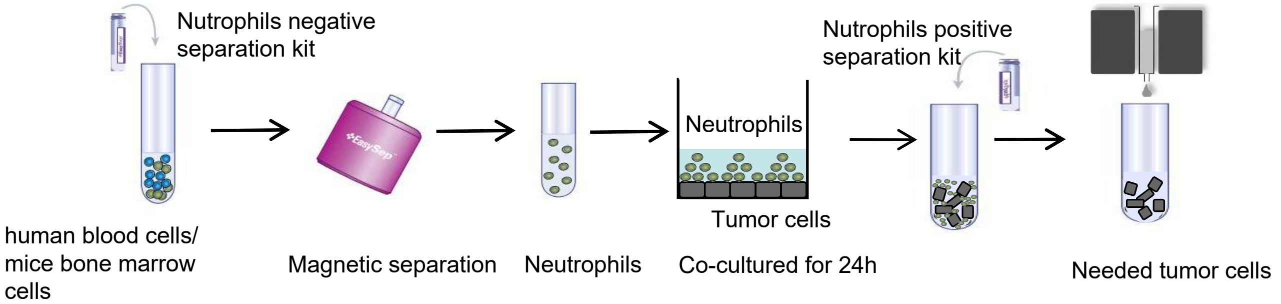

B

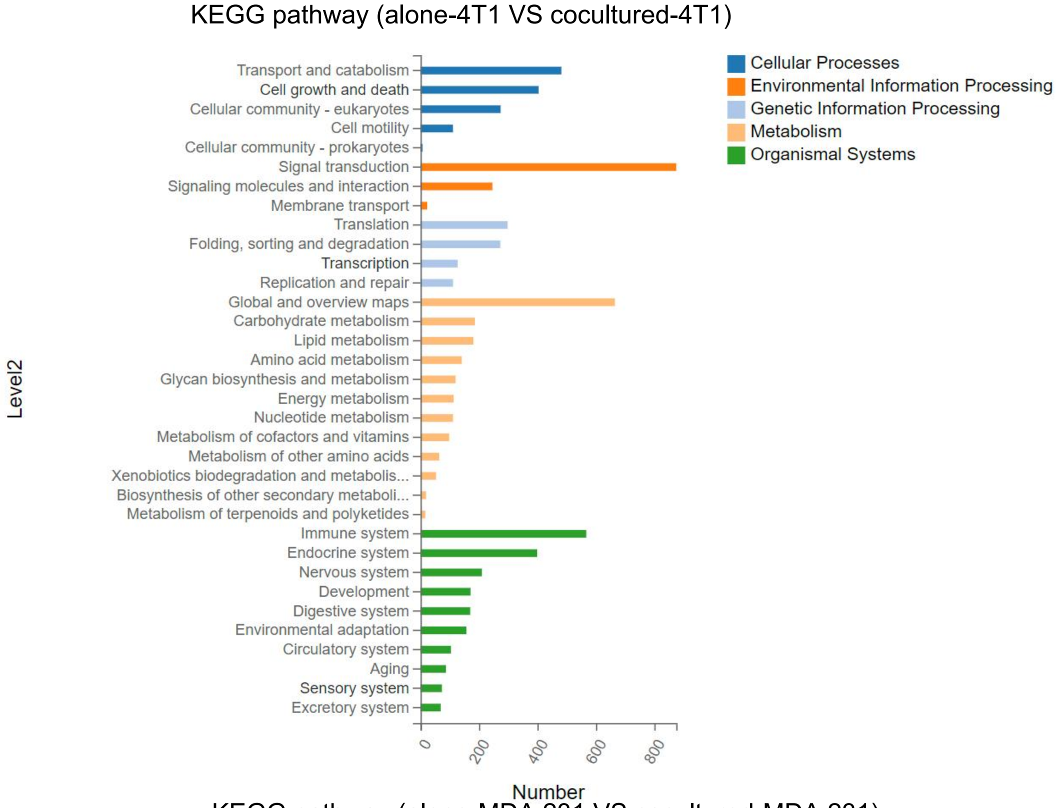

C

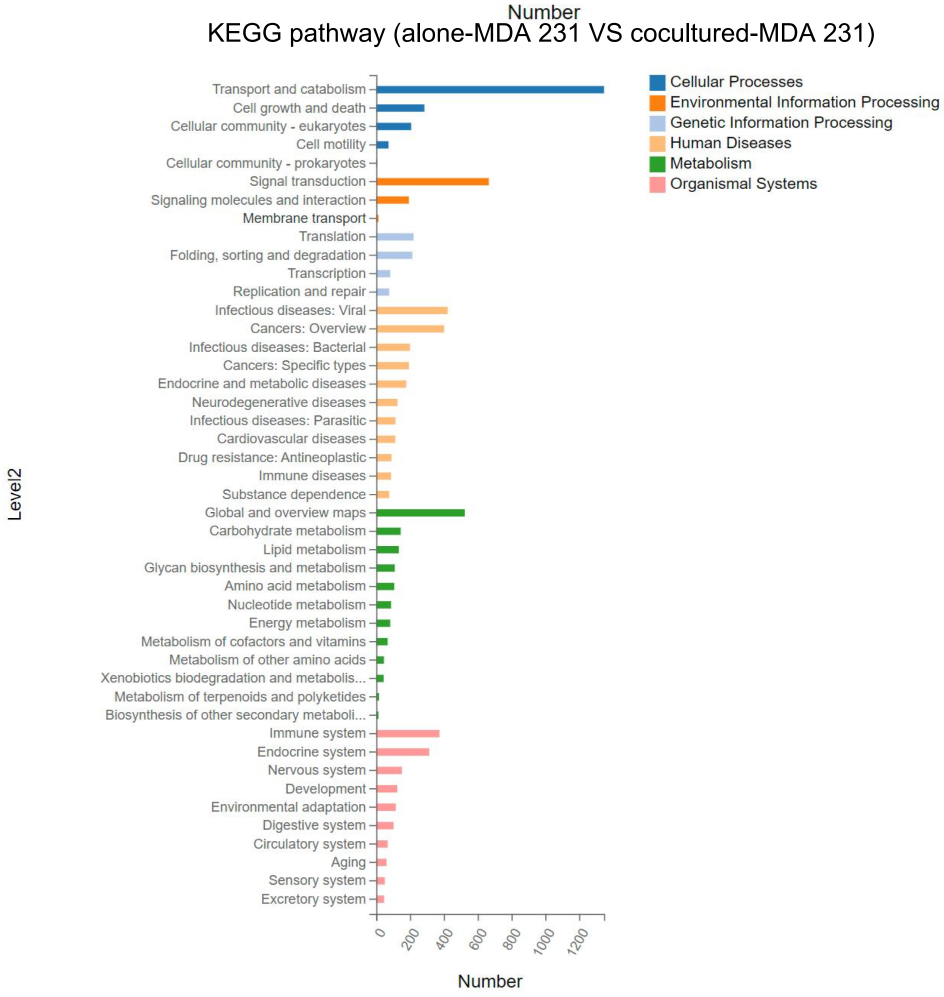

Figure S3. Related to Figure 4

(A) Schematic diagram of tumor cells sorting from co-cultured system in Figure 4G *in vitro*.

(B) KEGG pathway classification of differentially expressed genes between alone-4T1 and co-cultured-4T1.

(C) KEGG pathway classification of differentially expressed genes between alone-MDA-MB-231 and co-cultured-MDA-MB-231.

Figure S4. Related to Figure 4

A

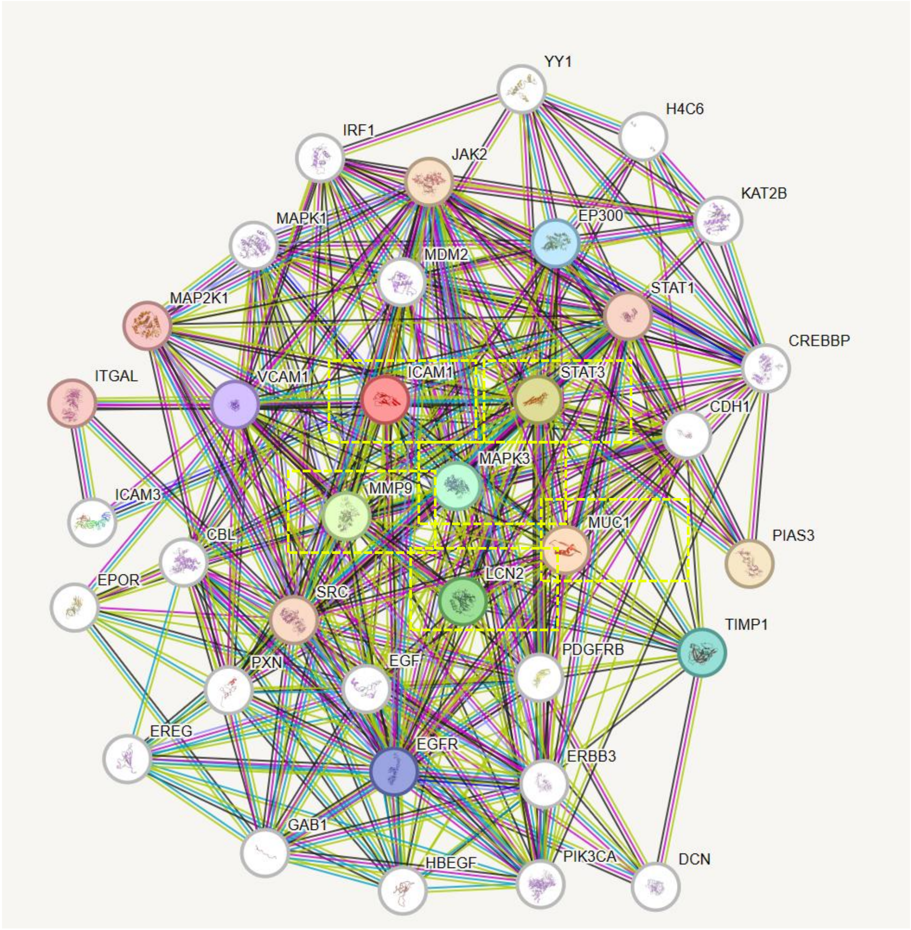

B

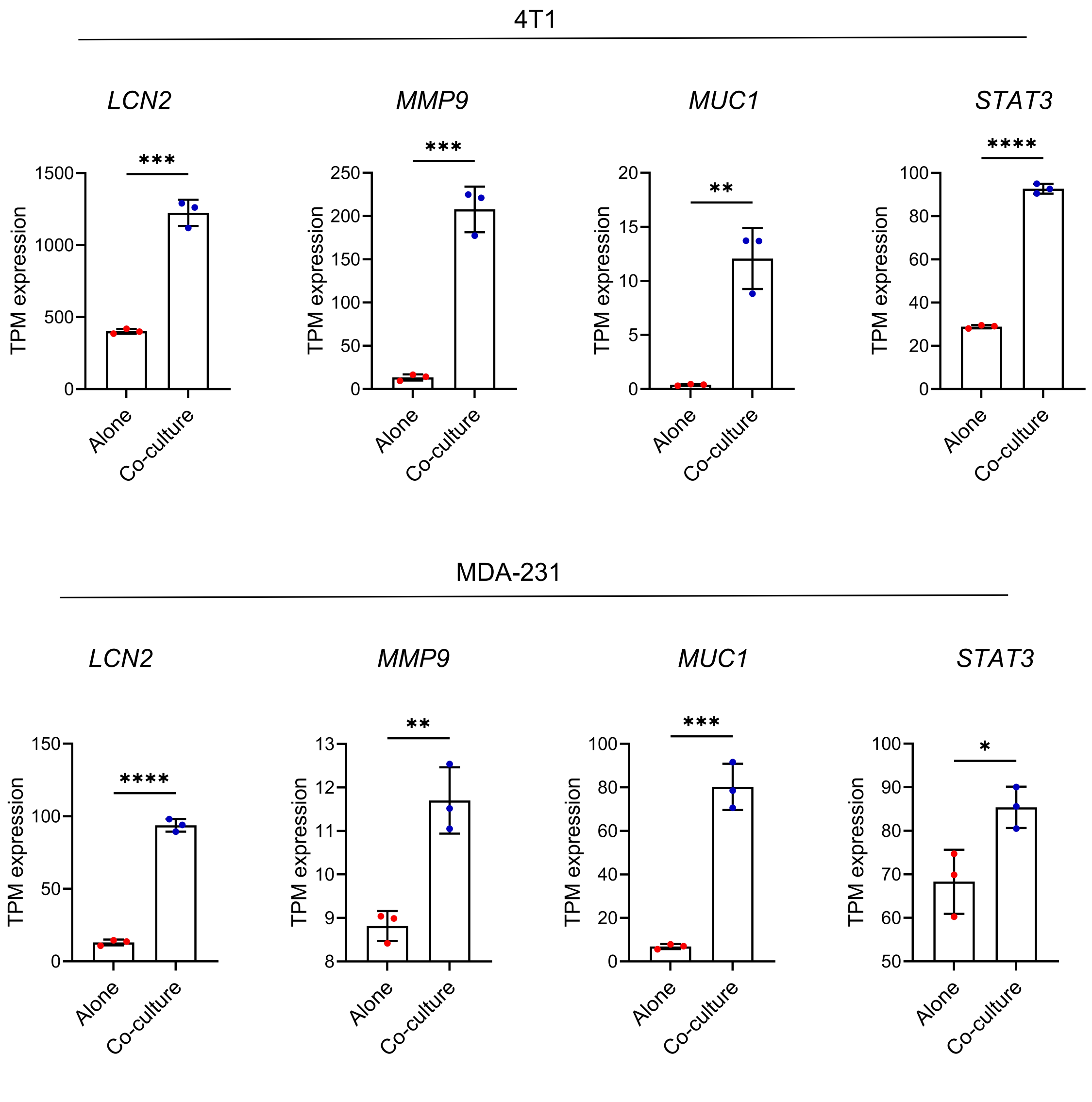

Figure S4. Related to Figure 4

(A) The protein interaction of MAPK pathway with differential genes of tumor cell lines co-cultured with or without neutrophils in STRING database.

(B) Analysis of the mRNA expression of *LNC2*, *MMP9*, *MUC1*, *STAT3* in 4T1 and MDA-MB-231 co-cultured with or without neutrophils.

Data are presented as the means ± SD from one representative experiment. Similar results were obtained from three independent experiments, unless indicated otherwise. Statistical analysis was performed by two-tailed unpaired Student's t test (B). ns, not significant, *p<0.05, **p<0.01, and ***p<0.001.

Figure S5. Related to Figure 5

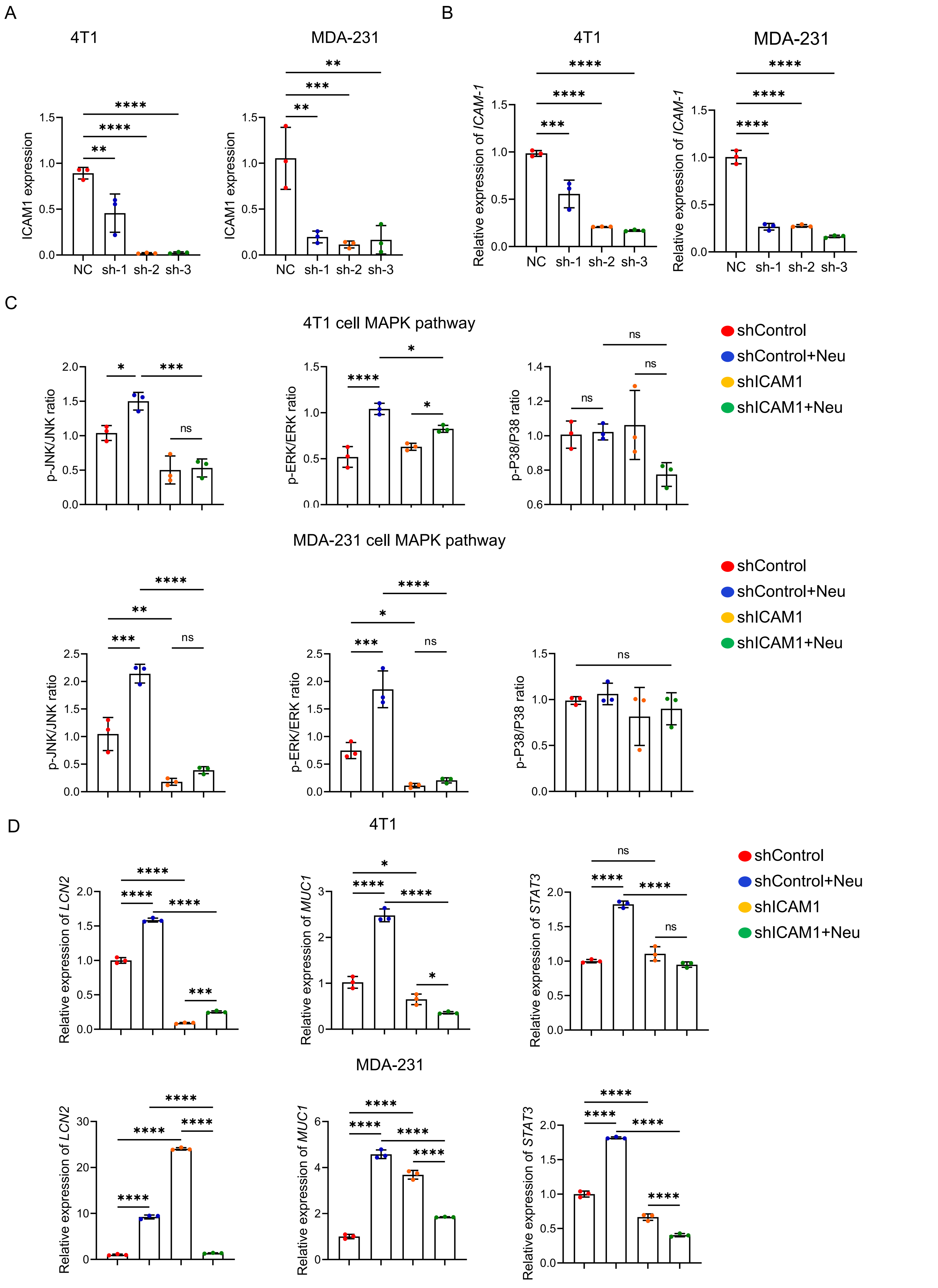

Figure S5. Related to Figure 5

(A) Quantitative analysis of ICAM1 protein in different tumor cell lines. *shControl*, tumor cells transfected with vector lentivirus. *shICAM1-1, shICAM1-2, shICAM1-3,* tumor cells transfected with *shICAM1* lentivirus of different target regions.

(B) Quantitative analysis of *ICAM1* expression in different tumor cell lines.

(C) Quantitative analysis of MAPK pathway protein in different tumor cell lines co-cultured with or without neutrophils.

(D) Analysis of the mRNA expression of *LNC2, MUC1, STAT3* in different tumor cell lines co-cultured with or without neutrophils.

Data are presented as the means ± SD from one representative experiment. Similar results were obtained from three independent experiments, unless indicated otherwise. Statistical analysis was performed by one-way ANOVA (A-D). ns, not significant, *p<0.05, **p<0.01, and ***p<0.001.

Figure S6. Related to Figure 5

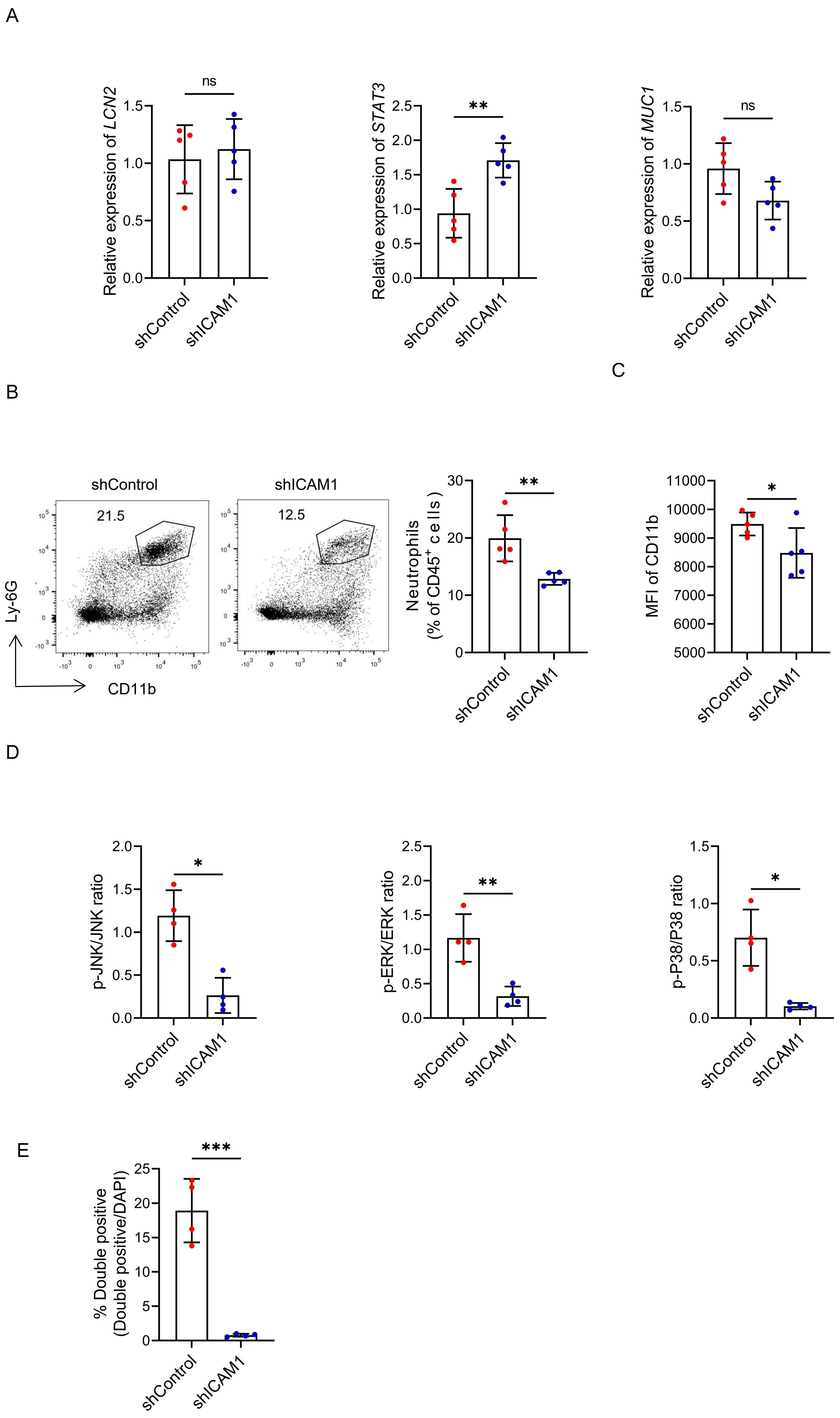

Figure S6. Related to Figure 5

(A) Analysis of the mRNA expression of *LCN2*, *STAT3* and *MUC1* in primary tumors from 4-week tumor bearing mice inoculated with *shControl*- or *shICAM1*-4T1 cells.

(B) Flow cytometry analysis and quantification of neutrophils in primary tumors from 2-week tumor bearing mice inoculated with *shControl*- or *shICAM1*-4T1 cells.

(C) Flow analysis of CD11b in tumor infiltrating neutrophils in primary tumors from 2-week tumor bearing mice inoculated with *shControl*- or *shICAM1*-4T1 cells.

(D) Quantitative analysis of MAPK pathway protein in primary tumors from 4-week tumor bearing mice inoculated with *shControl*- or *shICAM1*-4T1 cells.

Quantitative analysis of immunofluorescence staining of p-JNK$^+$MMP9$^+$ cells in primary tumors from 4-week tumor bearing mice inoculated with *shControl*- or *shICAM1*-4T1 cells.

Data are presented as the means ± SD from one representative experiment. Similar results were obtained from three independent experiments, unless indicated otherwise. Statistical analysis was performed by two-tailed unpaired Student's t test (A-E). ns, not significant, *p<0.05, **p<0.01, and ***p<0.001.

Figure S7. Related to Figure 6

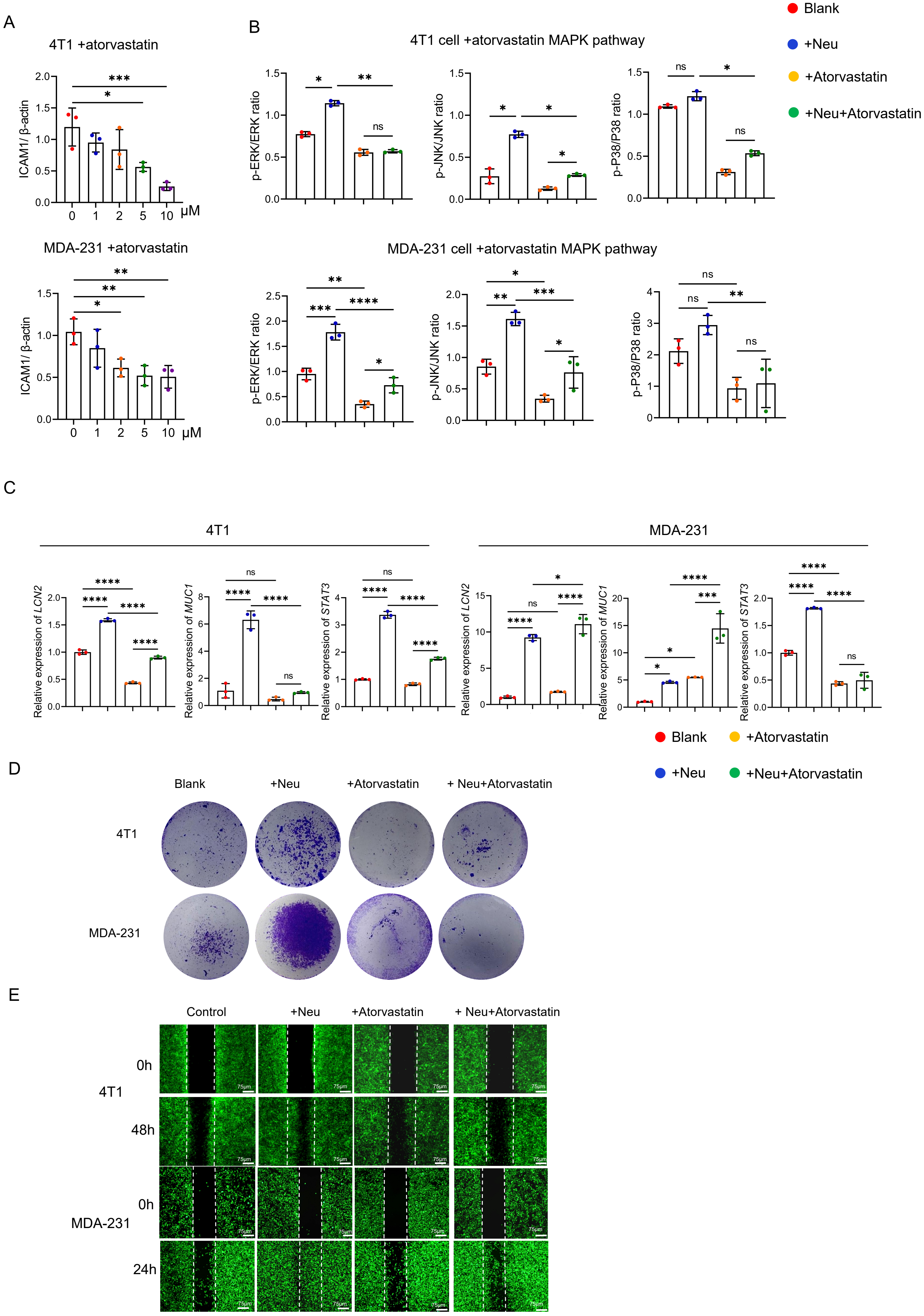

Figure S7. Related to Figure 6

(A) Quantitative analysis of the ICAM1 protein on tumor cell lines treated with atorvastatin for 24 hour *in vitro* in Figure 6A.

(B) Quantitative analysis of the MAPK pathway protein in tumor cell lines co-cultured with or without neutrophils in the presence or absence of atorvastatin (4T1 10 μM, MDA-MB-231 5 μM) for 24 hours *in vitro* in Figure 6B.

(C) Quantitative analysis the mRNA expression of *LNC2, MUC1, STAT3* in tumor cell lines co-cultured with or without neutrophils in the presence or absence of atorvastatin for 24 hours *in vitro*.

(D) Matrigel invasion assay images of tumor cell co-cultured with or without neutrophils in the presence or absence of atorvastatin for 24 hours *in vitro* in Figure 6D.

(E) Migration capability images of tumor cell lines co-cultured with or without neutrophils in the presence or absence of atorvastatin for 24 hours *in vitro* in Figure 6E.

Data are presented as the means ± SD from one representative experiment. Similar results were obtained from three independent experiments, unless indicated otherwise. Statistical analysis was performed by one-way ANOVA(A-C). ns, not significant, *$p<0.05$, **$p<0.01$, and ***$p<0.001$.

Figure S8. Related to Figure 6

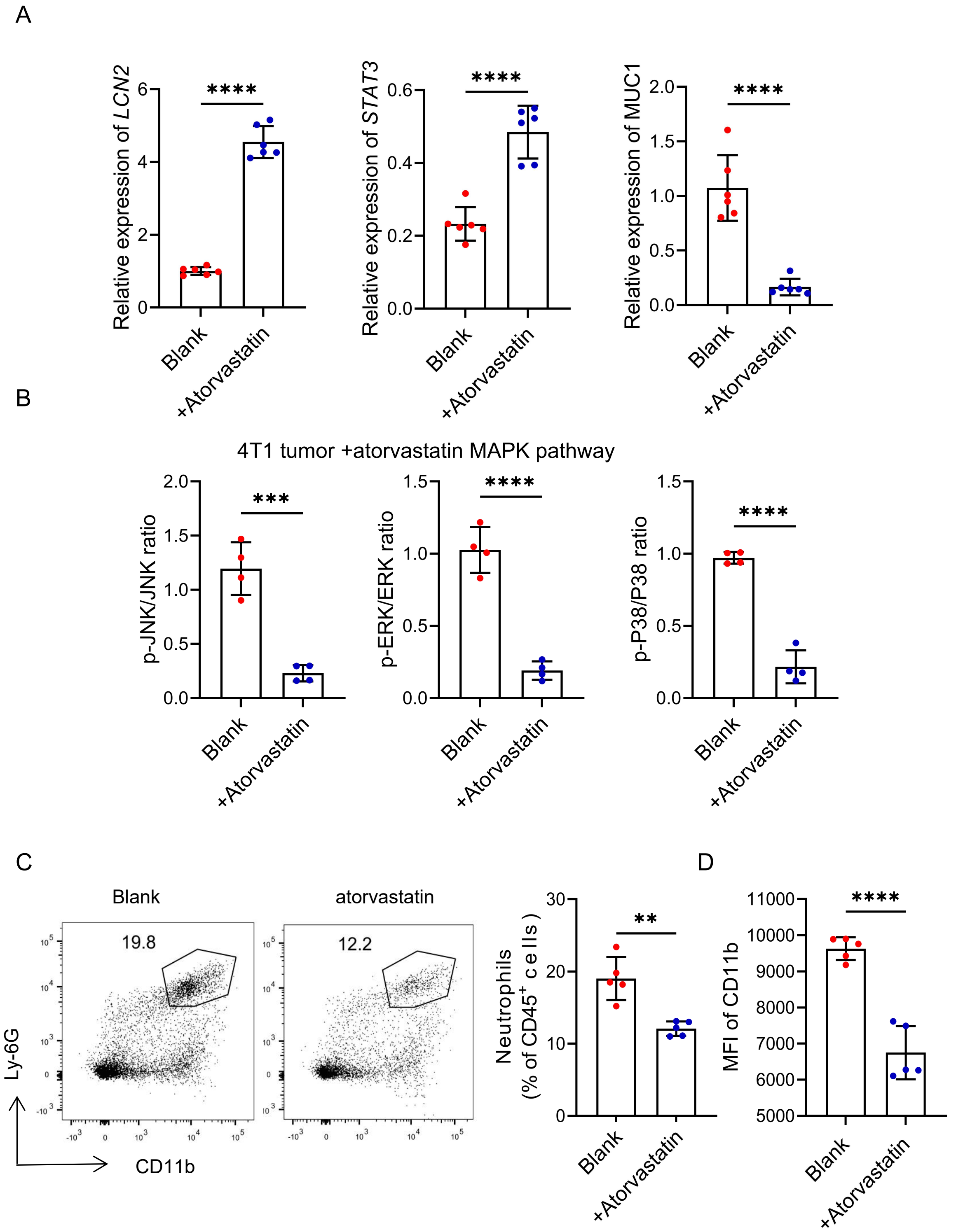

Figure S8. Related to Figure 6

(A) Analysis of the mRNA expression of *LCN2*, *STAT3* and *MUC1* in primary tumors from 4-week tumor bearing mice inoculated with 4T1 cells after th intragastric administration of atorvastatin or PBS.

(B) Quantitative analysis of the MAPK pathway protein in primary tumors from 4-week tumor bearing mice inoculated with 4T1 cells after the intragastric administration of atorvastatin or PBS in Figure 6H.

(C) Flow cytometry analysis and quantification of neutrophils in primary tumors from 2-week tumor bearing mice inoculated with 4T1 cells after the intragastric administration of atorvastatin or PBS.

(D) Flow analysis of CD11b in tumor infiltrating neutrophils in primary tumors from 2-week tumor bearing mice inoculated with 4T1 cells after the intragastric administration of atorvastatin or PBS.

Data are presented as the means ± SD from one representative experiment. Similar results were obtained from three independent experiments, unless indicated otherwise. Statistical analysis was performed by two-tailed unpaired Student's t test (A-D). ns, not significant, *p<0.05, **p<0.01, and ***p<0.001.
